# Oxygen Binding Kinetics and Coordination States of Hemoglobins from Early Land Plants

**DOI:** 10.1021/acsomega.5c09460

**Published:** 2025-11-27

**Authors:** Sydney Dvorak, Jonathan D. Monroe, Kenneth Hanson, Ryan Sturms

**Affiliations:** † Department of Chemistry and Physics, Drake University, Des Moines, Iowa 50311, United States; ‡ Department of Chemistry and Biochemistry, 2948 James Madison University, Harrisonburg, Virginia 22807, United States; § Department of Chemistry & Biochemistry, 7823 Florida State University, Tallahassee, Florida 32306, United States

## Abstract

Bryophytes, comprising mosses (*Bryophyta*), hornworts (*Anthocerotophyta*), and liverworts (*Marchantiophyta*), represent early diverging lineages in land plant evolution. Each of these phyla contains hemoglobin genes whose functional properties remain largely unexplored. Here, we report phylogenetic analysis that confirms that bryophyte globins form a distinct monophyletic group equally distant from both class 1 and class 2 nonsymbiotic hemoglobins of vascular plants. Spectroscopic characterization revealed that all three representative bryophyte hemoglobins exhibit predominant hexacoordination in the ferrous deoxy state. This predominantly hexacoordinate structure and the auto-oxidation rates, which are similar to other nonsymbiotic hemoglobins, are inconsistent with efficient oxygen transport function. However, kinetic analysis revealed a striking paradox: oxygen binding and dissociation rates closely resembling those of oxygen-transporting leghemoglobins. These findings reveal that bryophyte globins possess the kinetic capacity for rapid oxygen exchange but have structural features, namely, a hexacoordinate heme prosthetic group, that preclude efficient oxygen transport. This suggests that hemoglobins from early land plants served alternative physiological functions and that the inherent oxygen-binding capabilities of the globin fold were later optimized for transport in vascular plant lineages.

## Graphical abstract

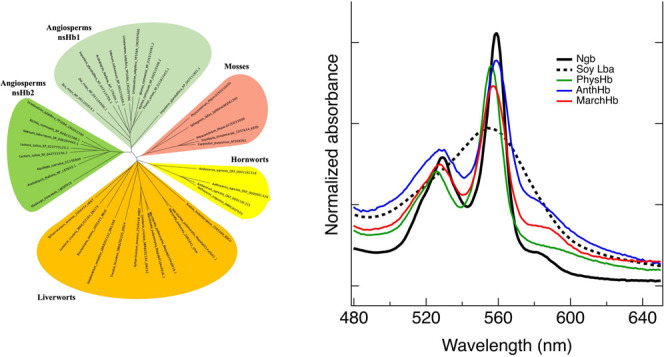

## Introduction

Hemoglobin (Hb) proteins are found across all three domains of life: archaea, bacteria, and eukarya.^1,2^ Among eukaryotes, Hbs have been found in both plant and animal lineages.^2–4^ The first plant Hb to be discovered was the symbiotic leghemoglobin, with nonsymbiotic and truncated hemoglobin being discovered later.^5^

Previous phylogenetic studies indicate that these plant Hbs have evolved into distinct monophyletic groups.^1,2,4–7^ These include leghemoglobins (Lbs), class 1 and 2 nonsymbiotic hemoglobins (nsHbs), and class 3 truncated Hbs. Class 3 truncated Hbs are structurally distinct globins with a 2/2 helical fold rather than the canonical 3/3 fold observed in full-length globins.^8^ Furthermore, these analyses have placed bryophyte globins, which are sometimes categorized as class 0 Hbs, distinct from both class 1 and class 2 nsHbs.^6^

From a functional viewpoint, several decades of work have provided a thorough in vitro analysis of ligand binding and reaction kinetics to understand the varied roles that Hbs play.^3,9–20^ The oxygen transport function of symbiotic plant Hbs, the leghemoglobins, has been studied in-depth, specifically in their interactions with *Rhizobium* bacteria in nitrogen-fixing organisms.^21^ These Hbs are known to facilitate the diffusion of oxygen in infected root nodule cells in support of symbiotic nitrogen fixation.^22^ The nsHbs, however, do not primarily function to facilitate the diffusion of oxygen.^23^ Instead, nsHbs have a wide range of hypothesized functions, likely related to nitric oxide (NO) homeostasis, including abiotic stress response,^24^ NO signaling and scavenging,^16,25–27^ promoting plant development and symbiosis,^27–30^ and supporting anaerobic metabolism by functioning as an alternative oxidase.^15,25,28,31^ The physiological significance of these biochemical functions and their relative importance under different conditions remain active areas of research.

The evolutionary history of plants and their hemoglobins is an important context for understanding other physiological roles that Hbs may have. Extensive work has been done to develop a picture of the evolution of hemoglobins in plants, though much of this work focuses primarily on vascular plants.^2–4,6,22^ These analyses reveal distinctions between class 1 and class 2 nsHbs, the development of leghemoglobins from the class 2 nsHb lineage, and the categorization of bryophyte Hbs as Hb0 ^6^ based on sequence homology, gene structure, and their presence in early diverging plant lineages. This predicted phylogeny follows the expected evolutionary course of land plants;^4,32^ however, little work has been done to understand the function of early land plant hemoglobins.

The bryophytes, made up of mosses, hornworts, and liverworts, are known to have diverged early in the evolution of land plants.^32,33^ Each of these lineages can be used to gain insight into ancient functions of green plant proteins because they display low rates of chromosomal evolution, whole genome duplication, and polyploidy, all advantageous traits for studying functions of conserved proteins in early land plants.^33,34^

All three bryophyte lineages, mosses, hornworts, and liverworts, contain Hb genes that are related to each other and other nsHbs, as shown in Figure 1. Despite their evolutionary significance, the kinetic properties and ligand binding characteristics of bryophyte Hbs remain largely uncharacterized, with only limited data available on oxygen binding capability through spectral analysis^35^ and no comprehensive comparison across the major bryophyte groups. Ligand binding measurements and spectral studies presented here provide the means to compare Hbs from moss (*Physcomitrella patens*), hornwort (*Anthoceros punctatis*), and liverwort (*Marchantia polymorpha*) to other Hbs of later developed plants (rice nsHb 1 and soybean Lba). This comparison gives insight into whether the biochemical properties of plant hemoglobins have been conserved throughout evolution and provides a platform for further in vitro and in planta studies to decipher the physiological roles of nsHbs.

**Figure 1:**
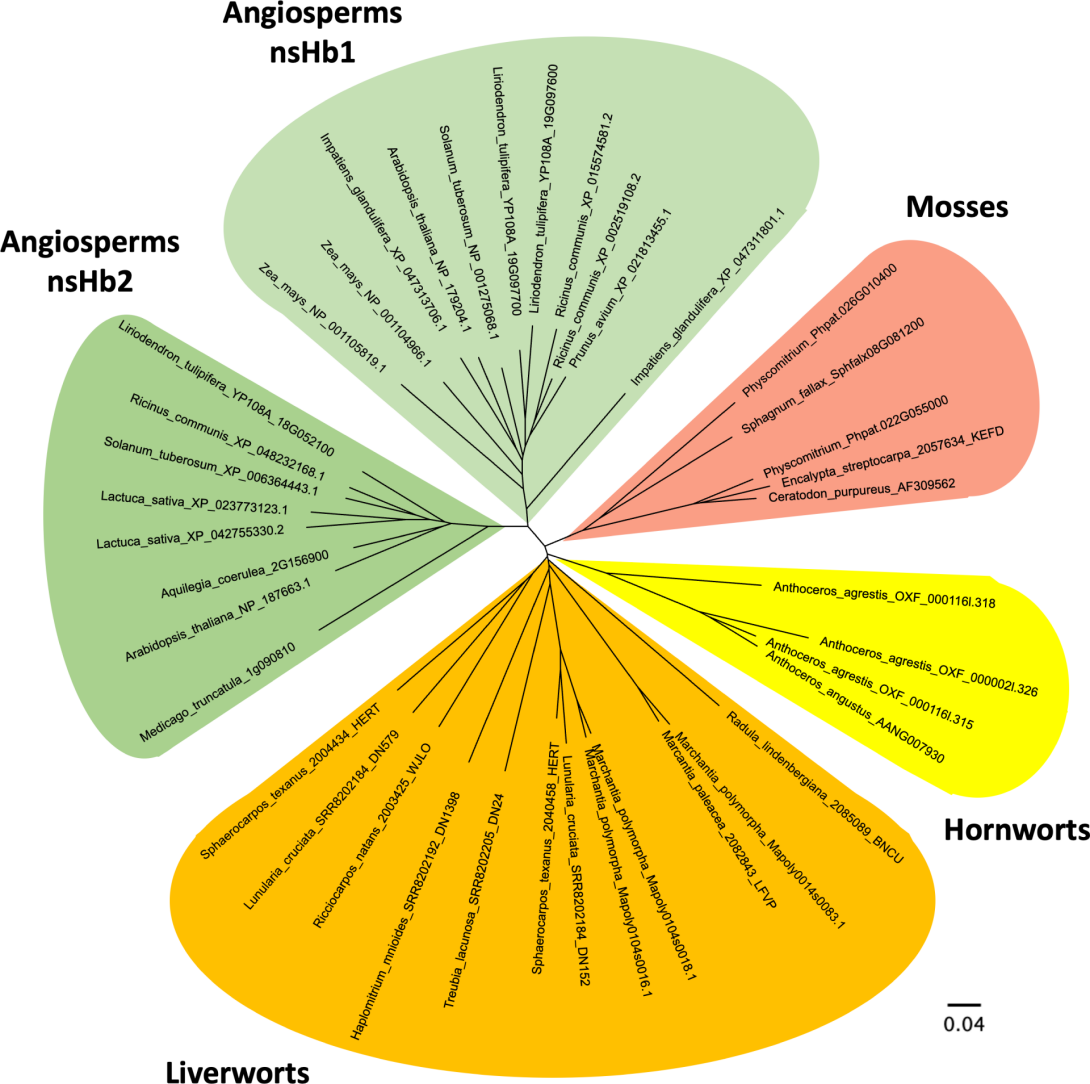
Radial phylogenetic tree of hemoglobins from viridiplantae and angiosperms, classes 1 and 2. The scale bar represents 0.04 substitutions per site. See Supporting Information for a complete list of species included in this analysis.

## Methods

### Protein Sequences

Amino acid sequences for the hemoglobins from *P. patens* and *M. polymorpha* were accessed in GenBank. The amino acid sequence for *Anthoceros punctatus* hemoglobin was identified by searching the *A. punctatus* proteome^36^ using blastP on the Galaxy server^37^ with *Physcomitrella* and rice nsHb1 sequences as queries. A single translated protein of 170 amino acids was found that shares approximately 50% identity with other plant hemoglobins, with E-values less than 1 × 10^–40^. Each of these three amino acid sequences was codon optimized for expression in *E. coli*. Genes were synthesized and subcloned into pET 28 vectors to include N terminal 6x His tags and the N terminal methionine of each gene by Genscript (Piscataway, New Jersey). These plasmids were then transformed into chemically competent BL21 (DE3) *E. coli* cells (Thermo Fisher Scientific; Waltham, MA) following manufacturer’s guidelines^38^ and labeled according to Table 1.

**Table 1:** Strains Used in This Study

strain	genotype	GeneID
RS300	BL21/pET28 a (+)-6*x*His-PhysHb	AAK14807.1^a^
RS301	BL21/pET28 a (+)-6*x*His-AnthHb	G37350.t1^b^
RS302	BL21/pET28 a (+)-6*x*His-MarchHb	OAE30109.1^a^

a Is from GenBank ID.

b Is from Supporting Information sets 1 and 2 in Chatterjee et al. 2022.^36^

To build the phylogenetic tree, hemoglobin sequences from *Physcomitrium patens*, *M. polymorpha,* and *Arabidopsis thaliana* were used in BLASTp or TBLASTn searches to identify sequences at the NCBI RefSeq database,^39^ Phytozome,^40^ and at MarpolBase.^41^ Selected sequences were then aligned, and the phylogenetic tree was generated using Clustal Omega.^42^ The tree was then visualized using FigTree v1.4.1 ^43^ The alignment is available in Figure S1.

### Production of Proteins

5 mL starter cultures of strains RS300, RS301, and RS302 were grown overnight at 37 °C in lysogeny broth^44^ to saturation. 1 mL of the starter culture was used to inoculate 1 L of TB media^45^ in 2 L Erlenmeyer flasks supplemented with 1 mL of 50 mg/mL kanamycin per flask. The flasks were incubated at 37 °C while being shaken overnight (18–20 h) at 250 rpm without induction. The cells were then harvested using centrifugation (2000*g* for 8 min). The cells were lysed by using a C3 homogenizer (Avestin; Ottawa, Ontario, Canada). Clarified lysate was applied to nickel affinity chromatography (G Biosciences; St. Louis, Missouri), which was used to purify the protein in a single step. Hemoglobins bound to the nickel NTA resin were eluted with 100 mM phosphate buffer (pH 7) supplemented with 100 mM imidazole. Collected fractions were dialyzed into 100 mM phosphate buffer (pH 7) to remove imidazole and concentrated using Pall Corporation (Port Washington, New York) Centrifugal Devices. The purity of the protein after purification was assessed using spectrophotometric analysis of the Soret/280 ratios.^19^ Human neuroglobin (Ngb) and leghemoglobin A (*Glycine max*) proteins were expressed and purified, as described above.

### Spectra Collection

All absorbance spectra were measured using a Cary 50 spectrophotometer (Agilent Technologies; Santa Clara, California). Absorbance spectra of each hemoglobin were collected in the ferric, ferrous deoxy, oxy, and carbonmonoxy (CO) forms^46^ in 100 mM phosphate buffer, pH 7.0. Ferrous hemoglobins were produced by reducing ferric samples with excess sodium dithionite. Ferrous CO samples were produced by bubbling the ferrous deoxy sample with carbon monoxide for 5–10 s in a cuvette. Oxy-hemoglobin was produced by reducing ferric hemoglobin with dithionite, followed by desalting on a G-25 column equilibrated with sodium phosphate buffer saturated with air.

### Kinetic Experiments

Flash photolysis and stopped-flow rapid mixing experiments were used to measure CO and O_2_ association and O_2_ dissociation for PhysHb, AnthHb, and MarchHb. Oxygen dissociation measured by stopped flow was carried out according to the methods of Smagghe et al.^47^ Briefly, solutions of oxyhemoglobin were mixed in a 1:1 ratio with a phosphate buffer equilibrated with varied carbon monoxide concentrations in a BioLogic (Seyssinet-Pariset, France) SFM 400 reactor. Time courses of oxygen dissociation were followed by spectral change in the Soret band and monitored by using a BioLogic MOS-250 spectrophotometer. Igor Pro (Wavemetrics; Portland, Oregon) was used to fit the average of 10 replicates to a single exponential function.

### Transient Absorption

A 1.0 cm × 0.2 cm glass cuvette, sealed with a rubber septum, was used for all Transient Absorption (TA) measurements. To ensure sufficient light transmission for the measurements, each sample was kept optically dilute (O.D. < 0.5) at 538 nm. Special care was taken with the oxygen-containing samples to ensure they were prepared and measured within 1 h to minimize sample degradation. An Edinburgh LP980 laser flash photolysis spectrometer system equipped with a Continuum Horizon OPO and a Continuum Surelite EX Nd/YAG laser (5 ns IRF, operated at 1 Hz, beam diameter ∼0.5 cm, 2.5–5 mJ/pulse) was used as the excitation source when measuring the carbon monoxide samples. For the samples with oxygen, the traditional OPO setup was bypassed with a 532 nm fundamental; a nearly 3-fold increase in the excitation source power (35 mJ vs 95 mJ) was achieved. A pulsed 150 W Xe lamp was used to generate the white light probe; this light was passed through the sample and a TMS302-A monochromator (1800 grooves/mm grating) with a 300 mm focal length in Czerny-Turner configuration before being detected using a Hamamatsu R928 PMT. Detector outputs were processed using Edinburgh’s L900 (version 8.2.3, build 0) software package. Kinetic traces were then fit to a single exponential function with Igor Pro.

### Autooxidation

Measurements of autoxidation rates were carried out in triplicate according to the methods described in Brantley et al.^48^ and compared to other published rates.^48–51^ Oxygenated hemoglobin samples were prepared as described above. Once the protein eluted from the column, it was immediately placed in a cuvette and diluted so that the alpha and beta peaks read an absorbance between 0.2 and 0.4. The scanning kinetics program of the Cary 50 spectrophotometer was used to simultaneously collect absorbance spectra between 450 and 650 nm and time courses.

## Results and Discussion

### Phylogenetic Analysis

The “bryophytes” are composed of three plant lineages that diverged early in the land plant evolution. These lineages are the mosses, the hornworts, and the liverworts.^33,34^ Previous studies have identified hemoglobin genes in representatives of the moss (*P. patens*) and liverwort (*M. polymorpha*) lineages.^6^ However, little attention has been paid to globins in the hornwort lineage. To provide a broad comparison group consisting of globins from all three members of the bryophytes, a transcribed proteome from the hornwort, *Anthoceros punctatus* ^36^, was searched using the BLAST algorithm of the galaxy server using the genes from *Physcomitrella* and the gene for rice nsHb1 as query sequences. A single gene with approximately 50% identity to the query sequences was found in this proteome with an *E* value of less than 1 × 10^–40^.

This gene was aligned with other known plant hemoglobin genes (Figure S1) and displays key sequence characteristics of globin proteins, including proximal and distal histidine residues and a phenylalanine at position B10.^6^ Using this gene from *Anthoceros* along with the genes from *Physcomitrella* and *Marchantia,* BLAST searches were performed using Phytozome and MarpolBase to find globin genes in other nonvascular plants. These sequences were compiled and aligned (Figure S1) and used to construct the phylogenetic tree shown in Figure 1. This tree corroborates the placement of the bryophyte globins as a distinct monophyletic group that is equally distant to both class 1 and class 2 nonsymbiotic hemoglobins of flowering plants.

This phylogeny does not address the physical properties of the bryophyte globins that may be used to gain further insight into the role of the hemoglobin protein family in early evolved land plants. The in vitro experiments performed on each of these bryophyte hemoglobins were carried out to answer two outstanding questions related to the structure and function of these globins from ancient lineages: (1) What is the endogenous histidine coordination state of hemoglobins found in bryophytes? and (2) Do the kinetics of oxygen binding support a role in oxygen transport?

### Spectral Analysis

#### Coordination in Ferric and Ferrous Oxidation States

Oxygen transport hemoglobins are typified by a pentacoordinate heme iron, contrasting with the hexacoordinate heme iron observed in the nonsymbiotic hemoglobins (see ref 3 for a detailed description of these coordination states). Examination of the visible absorbance spectra in both ferric and ferrous oxidation states indicates the coordination state based on the spectral features present,^49,52,53^ as described below. Figure 2 shows the visible absorbance spectra for each bryophyte Hb in the ferric (panel A) and ferrous deoxy (panel B) states, as well as for human neuroglobin (Ngb; 100% hexacoordinate) and soybean Lba (Soy Lba; 100% pentacoordinate) as comparisons.

**Figure 2:**
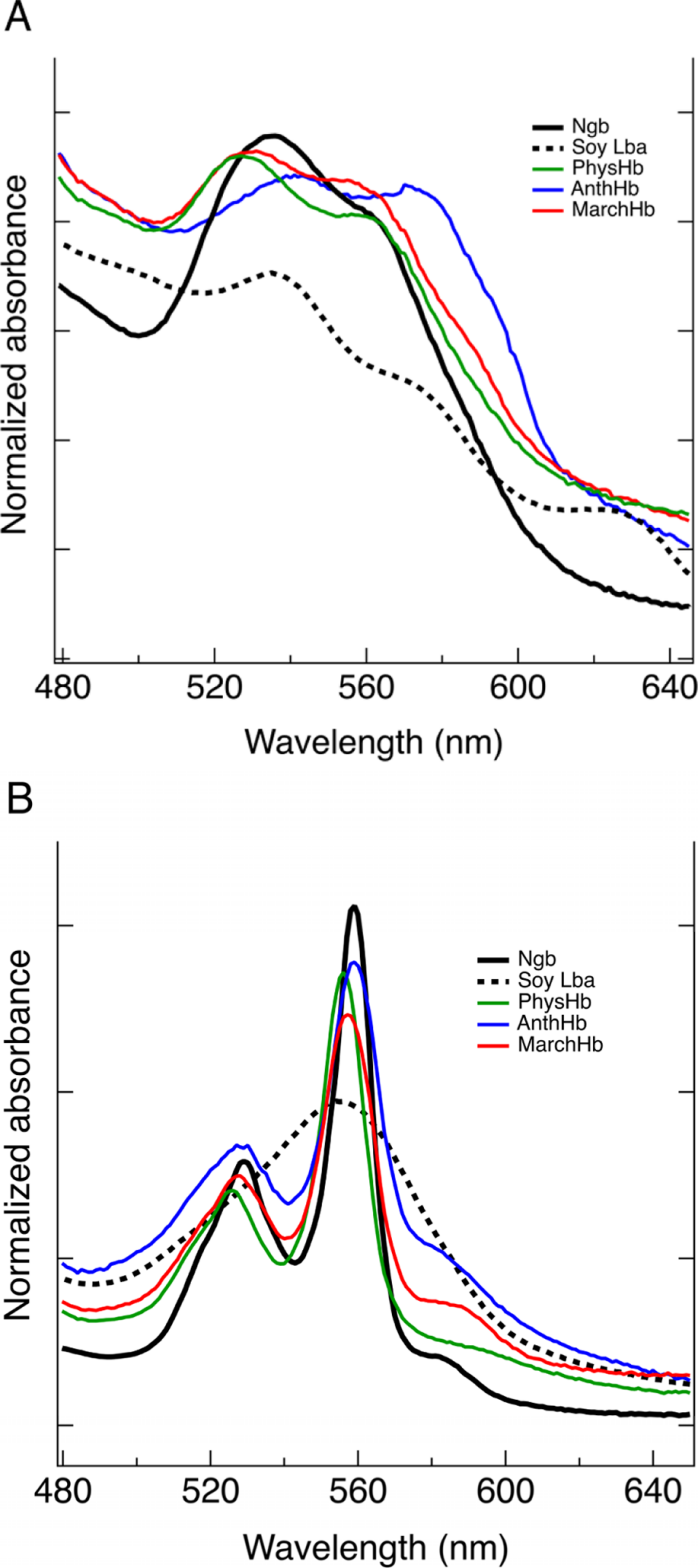
Ferric (A) and ferrous deoxy (B) absorbance spectra of bryophyte hemoglobins in 100 mM phosphate buffer pH 7.0: PhysHb (green), AnthHb (blue), and MarchHb (red), human neuroglobin (Ngb, solid black), and soybean leghemoglobin a (Soy Lba, dashed black).

In the ferric oxidation state at pH 7.0, all three bryophyte hemoglobins display absorbance at 530 nm and a shoulder at 560 (Figure 2). These features, similar to those observed in the characteristic visible spectrum of hexacoordinate neuroglobin^49^ (solid black trace), suggest that at equilibrium in the ferric state, bryophyte Hbs are predominantly hexacoordinate. In contrast, the broader, flatter absorbance over this range and the peak at 620 nm in the spectrum of Soy Lba (dashed black trace) are features observed in pentacoordinate ferric hemoglobins at pH 7.0.^52^ In the ferrous deoxy state (Figure 2B), hexacoordinate heme groups display splitting of the visible bands near 530 and 560 nm, as shown in the spectrum of Ngb (solid black trace), while ferrous deoxy pentacoordinate heme groups typically have a single, broad peak near 555 nm,^47^ as shown in the soybean Lba spectrum (dashed black trace). All three bryophyte globins have ferrous deoxy spectra that resemble ferrous Ngb, indicating that at equilibrium, hexacoordination is favored. Estimates of the equilibrium constants for hexacoordination (*K*_H_) by the distal histidine in these hemoglobins are provided in Table 2.

**Table 2:** Spectral Characterization of Ferrous Heme Coordination

protein	*A*_555_/*A*_540_	fraction hexacoordinate	*K*_H_
Ngb (Fully hexacoordinate, nonoxygen transport)	3.1	1	∼1000^47^
Lba (Fully pentacoordinate, oxygen transport)	1.2	0	0
Maize Hb1 (48% hexacoordinate, nonoxygen transport)	1.8	0.48	0.9^47^
PhysHb	2.7	0.83	∼4.8
MarchHb	2.1	0.53	∼1.1
AnthHb	2.0	0.50	∼1

The coordination state is critical for understanding the globin function. Among plant globins, symbiotic leghemoglobins exhibit pentacoordination. In contrast, nsHbs typically display varying degrees of hexacoordination. Within the nsHbs, class 1 nsHbs show weak hexacoordination, and class 2 nsHbs are more strongly hexacoordinate.^23^ In pentacoordinate globin proteins, the distal histidine remains unbound to the heme, creating space for reversible oxygen binding with moderate affinity, ideal for oxygen transport functions.^23^ In hexacoordinate globins, however, the distal histidine coordinates directly to the heme group and must dissociate before oxygen can bind. This results in competition between endogenous histidine and exogenous oxygen. This competition results in slower rates of oxygen association due to the kinetic barrier imposed by the need for distal histidine dissociation.^47^

To quantify the fraction of nsHb exhibiting hexacoordination (*F*_H_) in the ferrous oxidation state in the bryophyte globins, a previously described empirical relationship between the ratio of absorbance peak at 555 nm to the absorbance trough at 540 nm (A_555_/A_540_) and the fraction of hexacoordinate heme was used.^47^ Knowing *F*_H_ allows for the calculation of the equilibrium constant for hexacoordination (*K*_H_) through the use of the relationship in eq 1.^54^

1 FH=KH(1+KH)

This ratio is shown for each bryophyte hemoglobin as well as for three hemoglobins (soybean Lba, maize Hb1, and human Ngb) representing 0, ∼50, and 100% hexacoordination in Table 2. From this analysis, it can be estimated that bryophyte globins range from 50 to 80% hexacoordinate in the ferrous oxidation state. This degree of hexacoordination observed in bryophyte globins, which resembles the degree of hexacoordination observed in many nonsymbiotic hemoglobins,^23^ argues against a primary role in oxygen transport and supports the hypothesis that oxygen transport represents a later evolutionary development among vascular plant globins.

### Oxygen Binding

The discovery of hemoglobins in bryophytes was accompanied by UV–vis analysis of the oxygen-bound forms of these globins.^35^ Subsequent investigations of other nonsymbiotic globins have led to many hypotheses for the functions of plant hemoglobins, which vary by evolutionary class and include oxygen transport among the leghemoglobins, but not in the nonsymbiotic globins.^6^ Phylogenetic analysis places the bryophyte Hb genes as equally related to both the class two nonsymbiotic hemoglobins, which gave rise to the symbiotic leghemoglobins, and to the class one nsHbs that are not involved in oxygen transport.^6^ Given this evolutionary history, oxygen binding kinetics, i.e., association and dissociation rates, were measured for all three bryophyte globin representatives, and association equilibrium constants were calculated for each.

### Oxygen Dissociation

Figure 3 shows the absorbance at 412 nm with respect to time for oxyHb samples following treatment with 1 mM carbon monoxide (CO). The change in absorption is indicative of oxygen displacement by CO.^55^ The observed rate constants (*k*_obs_) were obtained from a single exponential fit of the average of 10 replicates and range from 0.34 s^–1^ in PhysHb to 0.4 s^–1^ in both AnthHb and MarchHb. Oxygen dissociation rate constants (*k*_O_2__) were calculated from *k*_obs_ using eq 2

2 $$k_{o2}=k_{obs}\left(1+\frac{k_{o2}^{\prime }\left[o_{2}\right]}{k_{co}^{\prime }\left[co\right]}\right)$$

where *k*′_O_2__ and *k*′_O_2__ are the association rates of oxygen and carbon monoxide to the pentacoordinate deoxyHb intermediate, respectively, as determined by TA spectroscopy (vide infra).

**Figure 3:**
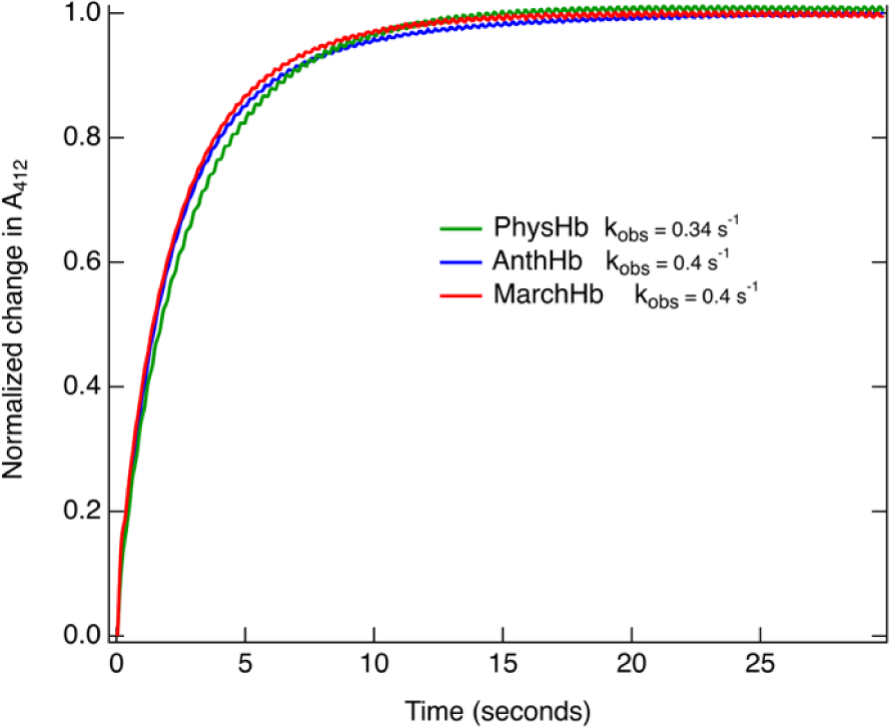
Normalized change in absorbance at 412 nm following the addition of 1 mM CO to each of the previously oxyHb samples.

### Oxygen Association Rate

Figure 4 shows the normalized TA kinetics at 420 nm for PhysHb, AnthHb, and MarchHb in air. Following excitation, the ground state bleach (i.e., ., negative ΔO.D.) and then return to baseline are consistent with rapid O_2_ dissociation (i.e., within the ∼10 ns instrument response function) followed by oxygen rebinding to the pentacoordinate deoxyHb intermediate^56^ The rebinding rate was determined using a single exponential fit and bimolecular rate constant ( $k_{O_{2},pent}^{\prime }$ ) determined using a value of 262 μM as the concentration of oxygen in air. The results are summarized in Table 3 along with rates for representative members from each phylogenetic class of plant hemoglobins. The rebinding rate of oxygen to the pentacoordinate deoxyHb in the bryophyte globins most closely resembles the rates observed for class 2 nsHbs.

**Figure 4:**
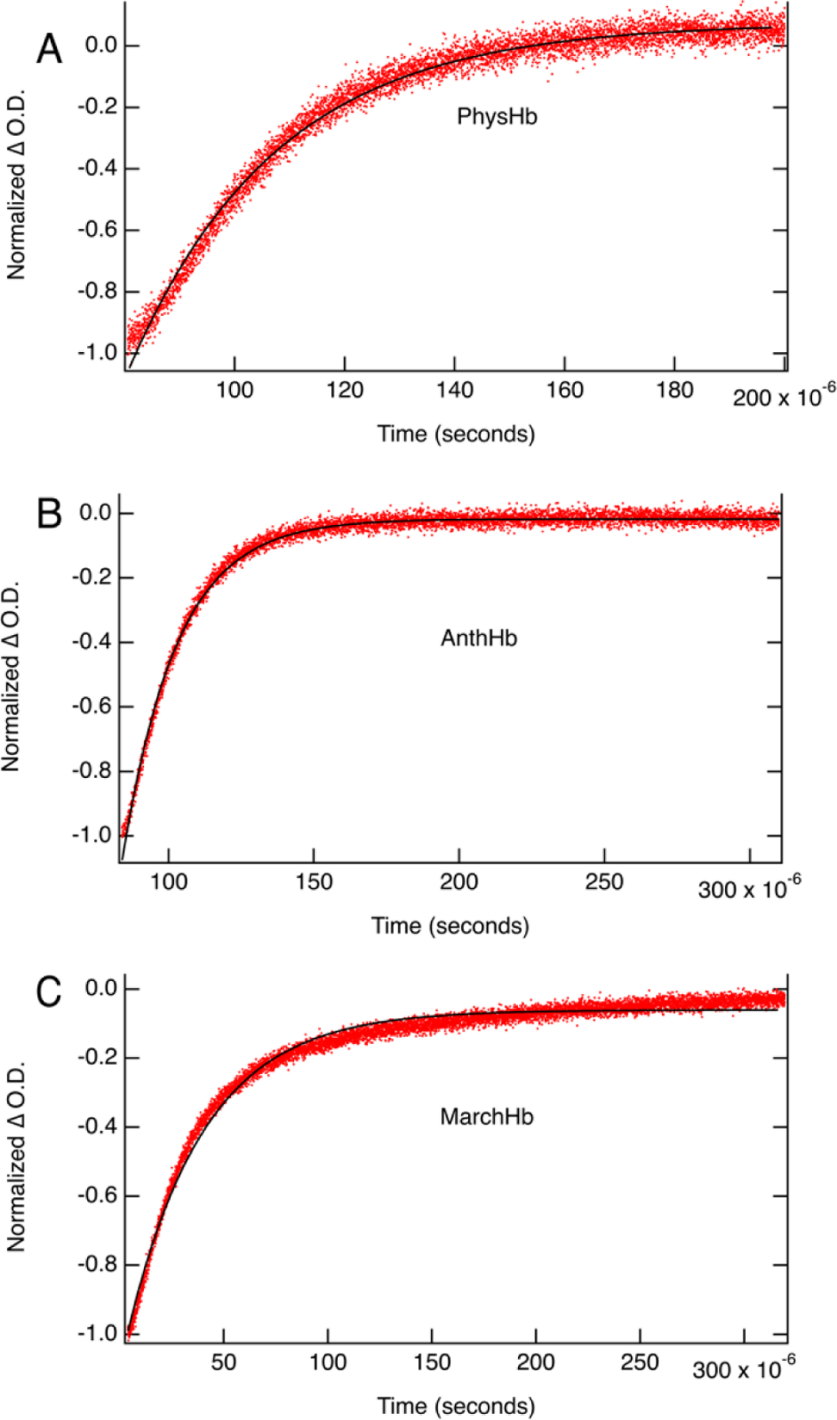
TA kinetics at 420 nm for PhysHb (A), AnthHb (B), and MarchHb (C) in aerated 100 mM phosphate buffer pH 7.0 (λ_ex_ = 532 nm). Black lines are single exponential fits to the kinetics.

**Table 3:** Oxygen Binding Parameters for Bryophyte and Angiosperm Hemoglobins

protein	$k_{O_{2\;},\;pent}^{\prime }$ (μM^–1^ s^–1^)	*k*_O_2__ (s^–1^)	$K_{O_{2},\;pent}$ (μM^–1^)	*K*_O_2__ (μM^–1^)	citation
PhysHb	148	5.2	28	4.8	this study
AnthHb	208	7.5	30	15	this study
MarchHb	102	6.9	13.6	6.5	this study
Class 1 nsHbs					
rice nsHb1	60	0.038	1600	540	^19^
*Arabidopsis* nsHb1	74	0.12	620	100	^23^
Lotus glb1-1	81	0.004	20,250		^57^
Class 2 nsHbs					
Lotus glb2	77	0.86	90		^57^
Arabidopsis nsHb2	150	2	75	2	^23^
Leghemoglobins					
Soy Lba	130	5.6	23	23	^58^

### Carbon Monoxide Association Rate

Dynamics of CO rebinding were monitored at 412 nm following excitation at 538 nm, resulting in photolysis of the bound CO. The results are shown in Figure 5A,C,E. The kinetics were fit with a single exponential function and yield bimolecular rate constants for PhysHb of 0.58 μM^–1^ s^–1^, for AnthHb of 3.34 μM^–1^ s^–1^, and for MarchHb of 2.05 μM^–1^ s^–1^. Additionally, we acquired the time-resolved TA difference spectra between carbonmonoxy Hb and ferrous deoxy Hb for PhysHb, AnthHb, and MarchHb in the presence of 1000 μM carbon monoxide. These results are shown in Figure 5B,D, and F. All three samples exhibit similar spectral features with excited state absorption 350–400 nm and 420–460 nm as well as a ground state bleach 400–420 nm (i.e., Soret band) and >450 nm (i.e., the Q-band). The similarity of the spectra over time and the presence of isosbestic points indicate the decay of a single species is being observed (i.e., dissociation and rebinding of CO). In contrast to the rates of oxygen rebinding that aligned more closely with the rates observed in class 2 nsHbs, these rates of CO rebinding to the pentacoordinate deoxyHb intermediate are more similar to the rates that are typically observed in class 1 nsHbs.

**Figure 5:**
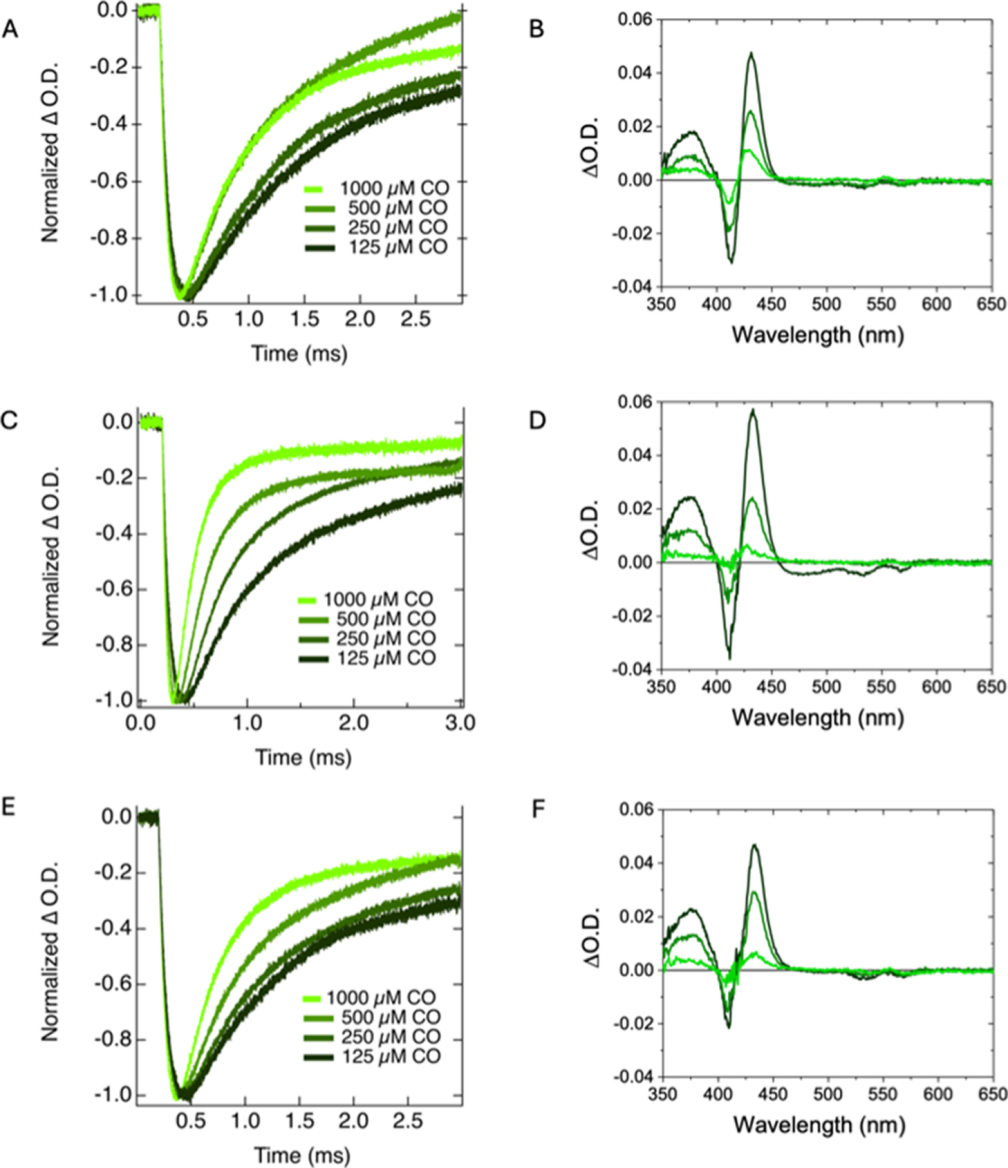
TA kinetics at 412 nm and varied CO concentrations (A,C,E) and difference spectra (B,D,F) of PhysHb (A,B), AnthHb (C,D), and MarchHb (E, F) in the presence of 1000 μM CO. Time slices progress from black to green at 500, 1500, and 2500 ms. (λ _ex_ = 538 nm).

### Oxygen Affinity

Using the association rate (*k*′_O_2__), as determined by flash photolysis, the oxygen dissociation rate (*k*_O_2__), as calculated using eq 2, and the K_H_ values determined from spectral analysis, the oxygen association equilibrium constant (*K*_O_2__) of each bryophyte globin was calculated using eq 3, and the results are summarized in Table 3.

3 $$K_{O_{2}}=\frac{K_{O_{2},\;pent}}{1+K_{H}}$$

Previously reported rates for association, dissociation, and affinity of other plant hemoglobin proteins from class 1, class 2, and the leghemoglobins are also included in the table for comparison. The oxygen on and off rates of the bryophyte globins, which were determined in this study, are comparable to those of class 2 nsHbs and leghemoglobins. However, the oxygen dissociation rates and oxygen affinity values differ from those of the class 1 nsHbs by at least an order of magnitude.

The oxygen binding kinetic analysis of bryophyte globin proteins revealed an intriguing evolutionary paradox. Despite exhibiting predominantly hexacoordinate structures that would preclude efficient oxygen transport, the transient pentacoordinate intermediates of these proteins display oxygen binding kinetics like known oxygen transporters, namely, the leghemoglobins. The measured oxygen dissociation rates of 5.2 s^–1^ (PhysHb), 7.5 s^–1^ (AnthHb), and 6.9 s^–1^ (MarchHb) closely match that of Soy Leghemoglobin (5.6 s^–1^), while the calculated *K*_O_2__ values of 28 μM^–1^ (PhysHb), 30 μM^–1^ (AnthHb), and 13.6 μM^–1^ (MarchHb) are comparable to Soy Lba (23 μM^–1^). This kinetic similarity extends to Class 2 hemoglobins, from which leghemoglobins evolved, suggesting the conservation of specific oxygen-binding parameters across plant evolution.

### Auto-Oxidation

Figure 6 depicts the change in absorbance for bryophyte globins during autoxidation. The absorbance at 575 nm is plotted versus time and fit to a single exponential decay, which gives the auto oxidation rate constant (*k*_ox_). The observed rate constants of autoxidation, shown in Table 4, for all three bryophyte globins are comparable to one another and to the nsHbs of *Arabidopsis* (0.5 h^–1^).^50^ However, the observed rates are significantly faster than those of myoglobin, a known pentacoordinate oxygen transport protein (0.055 h^–1^).^48^ The rate of autoxidation of Ngb, which is 100% hexacoordinate, is significantly faster (5.4 h^–1^)^49^ than the autoxidation rates determined here. These results are consistent with previous reports^48–50,59^ that correlate autoxidation rate to strength of hexacoordination. Further, these results suggest that the species studied here are not appropriate for roles that require a stably bound oxy–heme complex, including oxygen transport and NO scavenging via the NO dioxygenase reaction.

**Figure 6:**
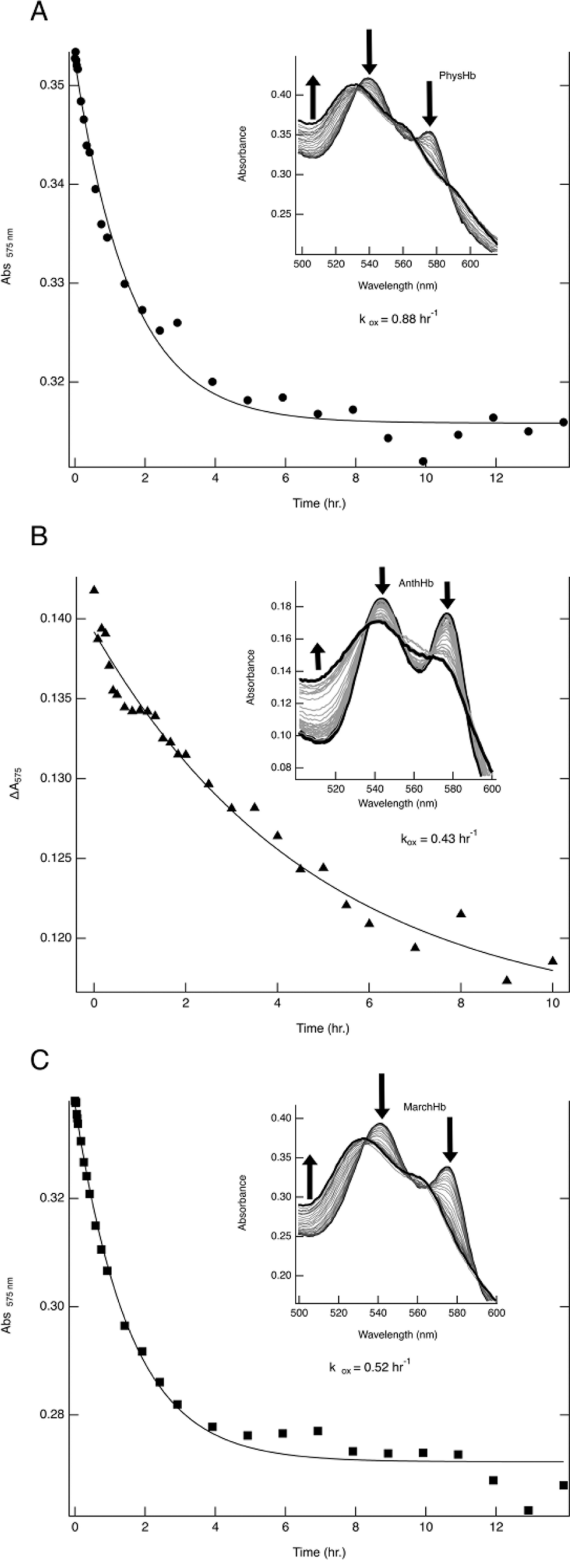
Absorbance change at 575 nm for (A): PhysHb, (B): AnthHb, and (C): MarchHb in 100 mM phosphate buffer, pH 7.0, during autoxidation. Insets show the measured absorbance spectra evolution over time.

**Table 4:** Rates of Auto-Oxidation pH 7.0, 25 °C

protein	*k*_ox_ (h^–1^)	*t*_1/2_ (min)	ref
PhysHb	0.88	47	this study
AnthHb	0.43	97	this study
MarchHb	0.52	80	this study
Mb^a^	0.055	756	^48^
At nsHb1	∼0.5	50	^50^
At nsHb2	∼2.97	14	^50^
Ngb †	5.4	11	^49^
rice nsHb1	0.08	520	^51^

a Collected at 37 °C. †Collected at pH 7.5.

## Conclusions

The biochemical characterization of bryophyte hemoglobins from moss (*P. patens*), hornwort (*A. punctatus*), and liverwort (*M. polymorpha*) presented here reveals an intriguing picture of globin functions in early diverging plants. The oxygen binding characteristics of bryophyte globins could kinetically support a role in oxygen transport; however, the predominantly hexacoordinate nature of these proteins argues against the interpretation of oxygen transport as a primary function. Instead, these findings suggest that bryophyte globins may represent a state in which the biochemical capacity for rapid oxygen exchange exists as a byproduct of the overall globin structure while serving alternative physiological functions. The coexistence of transport-compatible kinetics within a transport-incompatible distal histidine coordination points to roles that may occasionally require rapid oxygen association and dissociation such as oxidative stress protection, redox regulation, or oxygen sensing, roles that have been implicated in plant development and symbiosis formation.

In addition to providing insight into the oxygen binding capabilities of early land plants, these studies have clarified that the bryophyte globins form a distinct monophyletic grouping that is equally distant in sequence homology from both class 1 and class 2 nsHbs. We have also identified a likely target, *A. punctatus,* an early land plant that expresses a single 3/3 hemoglobin and forms symbiotic relationships with *Nostoc punctiforme*, that may serve as a model organism for in planta experiments to further study the nonoxygen transport functions of plant hemoglobins.

## Supplementary Material



## Notes

A species list of putative hemoglobin sequences used in the multiple sequence alignment and hemoglobin alignment used to construct a radial phylogenetic tree (PDF)

## Abbreviations

Hb: Hemoglobin
nsHb: nonsymbiotic hemoglobin
Lbs: leghemoglobins
Lba: leghemoglobin a
PhysHb: *Physcomitrella patens* hemoglobin
AnthHb: *Anthoceros punctatus* hemoglobin
MarchHb: *Marchantia polymorpha* hemoglobin
UV–vis: Ultraviolet–Visible
BLAST: Basic Local Alignment Search Tool
TB: Terrific Broth
Nd/Yag: Neodynium-doped Yttrium Aluminum Garnet
Xe: xenon
PMT: photomultiplier tube
TA: transient absorption
NO: nitric oxide.
