# Supplementary material for: Oxygen Binding Kinetics and Coordination States of Hemoglobins from Early Land Plants
Source: ACS Omega. 2025 Nov 27;10(48):59675–84. doi: 10.1021/acsomega.5c09460 (PMC12771424; doi:10.1021/acsomega.5c09460)
Supplement: Supplementary file 1 [file ao5c09460_si_001.pdf]

Oxygen Binding Kinetics and Coordination States of Hemoglobins from Early Land Plants.

*Sydney Dvorak[1], Jonathan D. Monroe[2], Kenneth Hanson[3], Ryan Sturms[1]**

AUTHOR ADDRESS

Sydney Dvorak[1] - Department of Chemistry and Physics, Drake University, Des Moines, Iowa 50311, United States

Jonathan D Monroe[2] – Department of Chemistry and Biochemistry, James Madison University, Harrisonburg, Virginia 22807, United States.

Kenneth Hanson[3] - Department of Chemistry & Biochemistry, Florida State University, Tallahassee, Florida 32306, USA

KEYWORDS. Hemoglobin, Bryophyte, Oxygen binding, Hexacoordination, Plant globin, Flash photolysis, Early land plants

Table S1. Species list

Clade	Species	Identifier	Source
Mosses	*Ceratodon purpureus*	AF309562	Phytozome
	Encalypta streptocarpa	2057634_KEFD	1000 Plants
	Physcomitrium patens	Phpat.022G055000	MarpolBase
	Physcomitrium patens	Phpat.026G010400	MarpolBase
	Sphagnum fallax	Sphfalx08G081200	Phytozome
Liverworts			
	Marchantia paleacea	2082843_LFVP	1000 Plants
	Marchantia polymorpha	*Mapoly0104s0018.1*	MarpolBase
	Marchantia polymorpha	Mapoly0104s0016.1	MarpolBase
	Marchantia polymorpha	Mapoly0014s0083.1	MarpolBase
	Radula lindenbergiana	2085089_BNCU	1000 Plants
	Ricciocarpos natans	2003425_WJLO	1000 Plants
	Sphaerocarpos texanus	2040458_HERT	1000 Plants
	Sphaerocarpos texanus	2004434_HERT	1000 Plants
	Treubia lacunosa	SRR8202205_DN24	1000 Plants
Hornworts	*Anthoceros agrestis*	OXF_000002l.326	MarpolBase
	Anthoceros agrestis	OXF_000116l.315	MarpolBase
	Anthoceros agrestis	OXF_000116l.318	MarpolBase
	Anthoceros angustus	AANG007930	MarpolBase
Angiosperms	*Aquilegia coerulea*	2G156900	Phytozome
	Arabidopsis thaliana	NP_187663.1	NCBI
	Arabidopsis thaliana	NP_179204.1	NCBI
	Impatiens glandulifera	XP_047313706.1	NCBI
	Impatiens glandulifera	XP_047311801.1	NCBI
	Lactuca sativa	XP_023773123.1	NCBI
	Lactuca sativa	XP_042755330.2	NCBI
	Liriodendron tulipifera	YP108A_18G052100	Phytozome
	Liriodendron tulipifera	YP108A_19G097700	Phytozome
	Liriodendron tulipifera	YP108A_19G097600	Phytozome
	Medicago truncatula	1g090810	Phytozome
	Prunus avium	XP_021813455.1	NCBI
	Ricinus communis	XP_002519108.2	NCBI
	Ricinus communis	XP_015574581.2	NCBI
	Ricinus communis	XP_048232168.1	NCBI
	Solanum tuberosum	XP_006364443.1	NCBI
	Solanum tuberosum	NP_001275068.1	NC*BI*
	Zea mays	NP_001104966.1	NCBI
	Zea mays	NP_001105819.1	NCBI

Table S2. Hemoglobin sequence alignment


```
                                          .......10........20........30........40........50........60
Anthoceros_agrestis_OXF_000116l.318        1 MPLIALLV-----TFGEEEGARSKCPKIL----------------------------ISS
Anthoceros_agrestis_OXF_000002l.326        1 ------------------------------------------------------------
Anthoceros_agrestis_OXF_000116l.315        1 ------------------------------------------------------------
Anthoceros_angustus_AANG007930             1 ------------------------------------------------------------
Radula_lindenbergiana_2085089_BNCU         1 MAGIAVPQ-----FRP----------------PAAGVGTTNATLPSRGSARL-SLGDTSR
Ricciocarpos_natans_2003425_WJLO           1 MASMNLSR-----TLATR---ANWCTTAALDSLRGSWSTTTATVTPQVSMRRSVVGSTVK
Marchantia_polymorpha_Mapoly0014s0083.1    1 ------------------------------------------------------------
Marcantia_paleacea_2082843_LFVP            1 ------------------------------------------------------------
Lunularia_cruciata_SRR8202184_DN579        1 MAYVNLAS-----RVATR---APFCTNTGRTTTSKVTTRAADD-----AF---TLPSIRS
Sphaerocarpos_texanus_2004434_HERT         1 MACVKFAS-----NVGAR---GSLMTPFDTCHPSRSSGAVYAI-----QFPLGALPSIRS
Marchantia_polymorpha_Mapoly0104s0018.1    1 ------------------------------------------------------------
Marchantia_polymorpha_Mapoly0104s0016.1    1 ------------------------------------------------------------
Lunularia_cruciata_SRR8202184_DN152        1 ------------------------------------------------------------
Sphaerocarpos_texanus_2040458_HERT         1 ------------------------------------------------------------
Medicago_truncatula_1g090810               1 ------------------------------------------------------------
Arabidopsis_thaliana_NP_187663.1           1 ------------------------------------------------------------
Aquilegia_coerulea_2G156900                1 ------------------------------------------------------------
Lactuca_sativa_XP_042755330.2              1 ------------------------------------------------------------
Lactuca_sativa_XP_023773123.1              1 ------------------------------------------------------------
Liriodendron_tulipifera_YP108A_18G052100   1 ------------------------------------------------------------
Solanum_tuberosum_XP_006364443.1           1 ------------------------------------------------------------
Ricinus_communis_XP_048232168.1            1 ------------------------------------------------------------
Physcomitrium_Phpat.026G010400             1 MSGSHS-----------K--VR--VTQTVACLLPGCVKQRILNF-----C---ALVVVSL
Physcomitrium_Phpat.022G055000             1 ------------------------------------------------------------
Encalypta_streptocarpa_2057634_KEFD        1 ------------------------------------------------------------
Ceratodon_purpureus_AF309562               1 ------------------------------------------------------------
Sphagnum_fallax_Sphfalx08G081200           1 ------------------------------------------------------------
Haplomitrium_mnioides_SRR8202192_DN1398    1 --------------MASR--IVATTTHSLPAGYGGCWSQSSESYG---AV---ARVSVSN
Treubia_lacunosa_SRR8202205_DN24           1 MAGVTAQALGLQTSMPFV--ISS------R-TVGGRTTTSRLDSSQKGVC---AINLRTN
Zea_mays_NP_001105819.1                    1 ------------------------------------------------------------
Impatiens_glandulifera_XP_047311801.1      1 ------------------------------------------------------------
Zea_mays_NP_001104966.1                    1 ------------------------------------------------------------
Impatiens_glandulifera_XP_047313706.1      1 ------------------------------------------------------------
Solanum_tuberosum_NP_001275068.1           1 ------------------------------------------------------------
Arabidopsis_thaliana_NP_179204.1           1 ------------------------------------------------------------
Liriodendron_tulipifera_YP108A_19G097700   1 ------------------------------------------------------------
Liriodendron_tulipifera_YP108A_19G097600   1 --------------MDVS--IPS------P-----ASKVSMLSYSPSRTC---NVK--IG
Ricinus_communis_XP_015574581.2            1 --------MILFNYISAT--LRS------TSAVLLSDATGIKKYSARDVS---NNYIIQR
Prunus_avium_XP_021813455.1                1 ------------------------------------------------------------
Ricinus_communis_XP_002519108.2            1 ------------------------------------------------------------


                                          61 ........70........80........90.......100.......110.......120
Anthoceros_agrestis_OXF_000116l.318       28 ADCMHGKHRATIHR--RSSAAAQQHRRPGGLRLAHCVH--------LPPPPTLF---GGL
Anthoceros_agrestis_OXF_000002l.326        1 --------------------------------------------------MTTFEGVGVA
Anthoceros_agrestis_OXF_000116l.315        1 -----------------------------------------------------MTTPDVP
Anthoceros_angustus_AANG007930             1 ---MTETRRYELGAWAASGVAVAVFGDPRDLCKVLCFSHSLFSVTQSTESMTTFEGVGVP
Radula_lindenbergiana_2085089_BNCU        39 SAY--HQSRSPALRWTSS----KAFGTQTF-LPERCPFASVHRG------KG----RQPW
Ricciocarpos_natans_2003425_WJLO          53 SSSSGENRRHLWLSSTHS----GVFGPSEL-IVR---FASTSSG------SGAEGSQPGK
Marchantia_polymorpha_Mapoly0014s0083.1    1 ------------------------------------------------------------
Marcantia_paleacea_2082843_LFVP            1 ------------------------------------------------------------
Lunularia_cruciata_SRR8202184_DN579       45 NNKLSDRIAALKLSSAHS----RGFGDAEL-FHS---FASIS---------HSNTSPANL
Sphaerocarpos_texanus_2004434_HERT        48 KVF---ARSTTALSSSQS----KTFGSIEF-FRC---FAATS---------GSEPSQGTR
Marchantia_polymorpha_Mapoly0104s0018.1    1 -----------------------------------------------------------M
Marchantia_polymorpha_Mapoly0104s0016.1    1 -----------------------------------------------------------M
Lunularia_cruciata_SRR8202184_DN152        1 ----------------------------------------------------------MV
Sphaerocarpos_texanus_2040458_HERT         1 ------------------------------------------------------------
Medicago_truncatula_1g090810               1 ------------------------------------------------------------
Arabidopsis_thaliana_NP_187663.1           1 -----------------------------------------------------------M
Aquilegia_coerulea_2G156900                1 ------------------------------------------------------------
Lactuca_sativa_XP_042755330.2              1 ------------------------------------------------------------
Lactuca_sativa_XP_023773123.1              1 ------------------------------------------------------------
Liriodendron_tulipifera_YP108A_18G052100   1 ------------------------------------------------------------
Solanum_tuberosum_XP_006364443.1           1 ------------------------------------------------------------
Ricinus_communis_XP_048232168.1            1 ------------------------------------------------------------
Physcomitrium_Phpat.026G010400            38 EEPT------------------------------M-ASS--I----GAAPVSVAPETAHG
Physcomitrium_Phpat.022G055000             1 ----------------------------------M-ASA--V----VNQSEAAAVRAPSK
Encalypta_streptocarpa_2057634_KEFD        1 ----------------------------------M-APA--I----VTPAQAATAPSPAK
Ceratodon_purpureus_AF309562               1 ----------------------------------M-APP--T----VG--TQAQAAPAMV
Sphagnum_fallax_Sphfalx08G081200           1 --------------------------------------------------------MAEV
Haplomitrium_mnioides_SRR8202192_DN1398   39 LRPSRCTGASYNVKWASS----VAFGNLKKVSAKQ-FGV-------TSSASANAKRGRLS
Treubia_lacunosa_SRR8202205_DN24          49 CTRTQPGSKSKSVSWSRT----AAFGDVNLVESSG-AYL-------TFQSRQSSIKDKYL
Zea_mays_NP_001105819.1                    1 ------------------------------------------------------------
Impatiens_glandulifera_XP_047311801.1      1 ------------------------------------------------------------
Zea_mays_NP_001104966.1                    1 -----------------------------------------------MA-----LAEADD
Impatiens_glandulifera_XP_047313706.1      1 ----------------------------------M-SQS-------SIP-----LSDGGN
Solanum_tuberosum_NP_001275068.1           1 ------------------------------------------------------------
Arabidopsis_thaliana_NP_179204.1           1 --------------------------------------------------------MESE
Liriodendron_tulipifera_YP108A_19G097700   1 -------------------------MDVSIPSAAL-TYS-------RVE-----KNGGGL
Liriodendron_tulipifera_YP108A_19G097600  29 FPPSSRLGHAREFSWRKT----EGFGDALSCRNAT-LIK-------SVE-----RNGGGL
Ricinus_communis_XP_015574581.2           42 NGPGLLPKRSLQLSWVRR----DGSAIELVCRNPK-VFT-------SVE-----RS-RGL
Prunus_avium_XP_021813455.1                1 -----------------------------------------------------------M
Ricinus_communis_XP_002519108.2            1 -------------------------------------------------------M-NTL
```

```
                                                 121 .......130.......140.......150.......160.......170.......180
Anthoceros_agrestis_OXF_0001161.318               75 SVRAFSPEQQVLVNESWKTMK-KDAGTVAVKFFLKIFEIAPTAKKLFSFIRDS-DVPLEK
Anthoceros_agrestis_OXF_0000021.326               11 TVGPLSPEKQALVNQSWEQMK-QDAPAVALKFFLKIFEIAPGAIALFSFLKDS-KVPLEQ
Anthoceros_agrestis_OXF_0001161.315                8 TVGPLAPEKQALVNQSWDQMK-QDAPGVALKFFLKIFEIAPGAIALFSFLKDS-KVPLEQ
Anthoceros_angustus_AANG007930                    58 TVGPLTPEKQALVSQSWEIMK-QDASAVALKFFLKIFEIAPGAIALFSFLKDS-KVPLEQ
Radula_lindenbergiana_2085089_BNCU                82 VVRAYTQEQAKLVKDSWKLVQKNDPGKHAVNFFVKIFEIAPTVKKMFSFMRDF-SGPMEK
Ricciocarpos_natans_2003425_WJLO                  99 VVAAYTREQAELVKSSWKLLK-KDAGKNGLTLFLKVFEIAPSVTQMFSFLRDS-SIPLEQ
Marchantia_polymorpha_Mapoly0014s0083.1            1 MDVNYSKAEAELVKASWEMFK-KDSIGNALIFFKKIFEVAPDMRDLFPFTQDY-TLPLEK
Marcantia_paleacea_2082843_LFVP                    1 MDVNYTKAQAELVKASWEMFK-KDSSGNALIFFTKIFEMAPDMRDLFPFTQDP-TLPLEK
Lunularia_cruciata_SRR8202184_DN579               88 TVRAYTPEQAELVKSSWQVLK-KDAGNHAHKFFLRVFEIAPSAAQLFSFLRDS-PVPIEQ
Sphaerocarpos_texanus_2004434_HERT                88 AVAEFTKEQAALVKETWKLLK-KDAGKNGMTLFLKVFEIAPSAQQLFSFLKDS-KVPLEE
Marchantia_polymorpha_Mapoly0104s0018.1            2 AASTFTKEQSQLVIESWQMVK-KDSATNSLTFFTKIFEIAPGARALFSFLKDS-DVPFDK
Marchantia_polymorpha_Mapoly0104s0016.1            2 AASTFTKEQSQLVIESWQMVK-KDSATHSVTFFAKIFEIAPGAKALFSFLKDS-DIPFDK
Lunularia_cruciata_SRR8202184_DN152                3 APPSFTVEQSQLVIESWQLLK-NDAAKHSLTFFTKIFEIAPGAKALFSFLKDS-SAPLDK
Sphaerocarpos_texanus_2040458_HERT                 1 -MASFTPEQSELVIESWQLLK-QDAATHSLTFFTKIFEIAPGAKALFSFLKDS-DAPLDK
Medicago_truncatula_1g090810                       1 -MDAFTERQEALVNSSWEAFKK-NIPQLSILFYTLILEKVPDAKDMFSFLKNFDGIP-HN
Arabidopsis_thaliana_NP_187663.1                   2 GEIGFTEKQEALVKESWEILKQ-DIPKYSLHFFSQILEIAPAAKGLFSFLRDSDEVP-HN
Aquilegia_coerulea_2G156900                        1 --MVFTEQQEALVKESWEIMKQ-NIPELSLQFFTTILEIAPAAKGLFSFLKDTDEVP-QN
Lactuca_sativa_XP_042755330.2                      1 --MSFSEKQEALVKEAWEIMKE-DITTLSLYFFTQVLEIAPAAKGLFSFLKDEEEIP-QD
Lactuca_sativa_XP_023773123.1                      1 --MGFSEKQEALVKESWEVMKE-DIPALSLHLYAMILEIAPEAKGLFSFLKDTNEIP-QN
Liriodendron_tulipifera_YP108A_18G052100           1 -MTTFTEQQEALVKESWEVMKL-NIPDLSLHFFTTILEIAPAAKGMFSFLKDSVEVP-QN
Solanum_tuberosum_XP_006364443.1                   1 --MGFTDKQEALVKDSWEFMKQDDIPQLSLRFFSLILEIAPAAKNMFSFLKDSNELP-QN
Ricinus_communis_XP_048232168.1                    1 --MRFSEKQEALVKESWEVMKQ-DIPHYSLRFFSIILEIAPAAKGMFSFLKDSDEIP-QS
Physcomitrium_Phpat.026G010400                    61 AEKVYSKESVALVKQSWELLKVD-AQANAVAFFKEVFEIAPAAKGFFSFMQDT-SIPFED
Physcomitrium_Phpat.022G055000                    20 PVKTYSKENEQLVKQSWEILKKD-AQRNGINFFRKVFEIAPGAKAMYSFLRDS-TIPFEE
Encalypta_streptocarpa_2057634_KEFD               20 PAREYSKANEQLVQQSWEILKKD-AQRNGINFFRKVFEIAPEAKAMYSFLRYS-TIPFEE
Ceratodon_purpureus_AF309562                      18 PARVYSKDDEVLVKKSWDILKKD-AQRNGINFFRKVFEIAPGAKSMYSFLRDS-TIPFEE
Sphagnum_fallax_Sphfalx08G081200                   5 PSSVFSKDSEILVKQSWELIKKD-TTTNAVIFFTNIFEIAPAAKDFFPFVKNS-TVPYEQ
Haplomitrium_mnioides_SRR8202192_DN1398           87 VTTAFTKEQSSLVKESWEALKKDNAGKHALKFFLKIFEIAPSVQRLFPFLRDS-SVPVEK
Treubia_lacunosa_SRR8202205_DN24                  97 VKNTFTKEQEQLVKKSWAALKKD-SSKHATDFFVRVFTIAPSAKRLFSFLRDS-SVPLDQ
Zea_mays_NP_001105819.1                            1 --MGFSEAQEELVLRSWKAMKSD-SESTALKFFLRIFEIAPGAKQMFSFLRDAGDAPLEK
Impatiens_glandulifera_XP_047311801.1              1 --MAFTEEDEALVLKSYDLMKLR-AGELALNFFLKIFEIAPAAKGLFSFLKDS-DLPLHQ
Zea_mays_NP_001104966.1                            9 GAVVFGEEQEALVLKSWAVMKKD-AANLGLRFFLKVFEIAPSAKQMFSFLRDS-DVPLEK
Impatiens_glandulifera_XP_047313706.1             14 MEVKFTEEQEALVVKSWSLMKKN-AGDLGLKLFLKIFEIAPSVKNMFSFLKDS-TIPVEQ
Solanum_tuberosum_NP_001275068.1                   1 -MSSFSEEQEALVVKSWGSMKKD-AGEWGLKFFLKIFEIAPSAKKMFSFLKDS-NVPLDQ
Arabidopsis_thaliana_NP_179204.1                   5 GKIVFTEEQEALVVKSWSVMKKN-SAELGLKLFIKIFEIAPTTKKMFSFLRDS-PIPAEQ
Liriodendron_tulipifera_YP108A_19G097700          23 KVRAFTEEQEALVVQSWNAMKKD-AAEVGLKFFLRIFEIAPSAKKLFSFLRDS-DVPLEK
Liriodendron_tulipifera_YP108A_19G097600          72 KVRAFTEEQEALVVKSWNVMRKD-AADLSLKFFLRIFEIAPSAKKLFSFLKDS-DVPVEK
Ricinus_communis_XP_015574581.2                   84 KVSAFTEEQEALVVKSWNSMKKN-AGELGVKFFLKIFEIAPSAKKLFTFLRDS-DVPVEQ
Prunus_avium_XP_021813455.1                        2 EGKVFTEEQETLVAKSWGVMKKN-SAELGLKFFLKIFEIAPSAQKLFSFLKDS-DIPLEK
Ricinus_communis_XP_002519108.2                    5 ECKAFTEEQEALVVKSWHVMKKN-SAELGLKFFLKIFEIAPSAKKLFSFLRDS-NIPVEQ
```

```
                                                 181 .......190.......200.......210.......220.......230.......240
Anthoceros_agrestis_OXF_0001161.318              133 NPQLKTHALLVLKTTGDAATQLGQKGALEAL-RPVLLELGRTHVGHGVIDEHFDVVKYAL
Anthoceros_agrestis_OXF_0000021.326               69 NPRLKVHALLVLKTFGEAATQLGKEGALDEL-TPVLQKLGRAHVPRGVIDPHFDVVKAAL
Anthoceros_agrestis_OXF_0001161.315               66 NPRLKVHALLVLKTFGEAATQLGKEGALEEL-TPVLQKLGRAHVPRGVIDPHFDVVKSAL
Anthoceros_angustus_AANG007930                   116 NPRLQVHALLVLKTFGEAATRLGKEGALDEL-TPVLQKLGRAHVPRGVIDPHFDVVKAAL
Radula_lindenbergiana_2085089_BNCU               141 NPKLKIHALQVFTLTGQSAAELSEKGGLNVL-LPKLMELGHSHADYGVLDEHFDVVKYAL
Ricciocarpos_natans_2003425_WJLO                 157 NSQLKAHALTVFKMVGDAAAQLGEKGGLDAL-RATLIDLGDSHLTYGVVNEHFDVVKYAM
Marchantia_polymorpha_Mapoly0014s0083.1           59 NYKLKAHALLVFKLTGDAAVQLGEHGSIESL-HPKLIDLGKKHLGYGVLLEHFVVVRTAL
Marcantia_paleacea_2082843_LFVP                   59 NYKLKAHALLVFKLTGDAAVQLGENGSIQSL-HPKLIDLGKKHLGYGVLLEHFVVVKSAL
Lunularia_cruciata_SRR8202184_DN579              146 NPHLKTHALIVFKLTGDAAAQLGEKGALDAL-RPKLVELGQSHILYGVLNEHFDVVKYSL
Sphaerocarpos_texanus_2004434_HERT               146 NVQLKGHALNVFKMTGDAAAQLGEKGALDAL-HPKLVELGQSHVVHGVMNEHFDVVKFAL
Marchantia_polymorpha_Mapoly0104s0018.1           60 NPKLKAHALNVFRLTGDSASMLGEKGAIDAL-HPKLKELGKKHVGYGVIAAHFDVVKSAL
Marchantia_polymorpha_Mapoly0104s0016.1           60 NPKLKAHALTVFRLTGDSACMLGEKGAIDAL-HPKLKELGKKHVGYGVIAAHFDVVKAAL
Lunularia_cruciata_SRR8202184_DN152               61 NPKLKAHALTVFRLTGDSASMLGEKGAIDAL-HPRLKELGRKHVGYGVIPAHFDVVKTAL
Sphaerocarpos_texanus_2040458_HERT                58 NPKLKAHALNVFRLTGDSASMLGEKGAIDAL-HPKLKLLGKKHVGYGVIAAHFDVVKTAL
Medicago_truncatula_1g090810                      58 NSTLEAHAELIFEMTRDSAVQLRAKGKVDVADDVTLEYLGSVHVQKGVIDLHFMVFKEAM
Arabidopsis_thaliana_NP_187663.1                  60 NPKLKAHAVKVFKMTCETAIQLREEGKVVVA-DTTLQYLGSIHLKSGVIDPHFEVVKEAL
Aquilegia_coerulea_2G156900                       57 NPKLKAHAVKVFKMTCEAAVQLREKGAVDLP-ESTLKYLGAVHVKKGVIDPHFEVVKEAL
Lactuca_sativa_XP_042755330.2                     57 NPKLKSHAVKVFKMTCEAAIQLREKGEVVVS-GSTLKYLGSVHLQKGIVDAHFAVVKEGV
Lactuca_sativa_XP_023773123.1                     57 NPKLKSHAVKVFKMVCESAIQLREKGEVVVS-GSSLKYLGSVHLQKGIVDPHFEVVKEAL
Liriodendron_tulipifera_YP108A_18G052100          58 NPKLKAHAVKVFKMTCESAVQLREKGEVVVA-ENTLKNLGSVHVKKGVVDAHFEVVKEAL
Solanum_tuberosum_XP_006364443.1                  58 NPKLKAHAVKVFKMTCESAIQLREKGEVVVG-ETTLKYLGSIHLQQGVADPHFEVVKEAL
Ricinus_communis_XP_048232168.1                   57 NPKLKAHAVKVFKMTCESAIQLREKGEVVVN-DTTLKYLGSVHVQKGVLDPHFEVVKEAL
Physcomitrium_Phpat.026G010400                   119 NPKVKFHALQVFKLTGDAAAQLGEKGAYELL-QSRLHSLAAKHLSKGVQDAHFEVVKEAL
Physcomitrium_Phpat.022G055000                    78 NPKVKNHARYVFMMTGDAAVQLGEKGAYQVL-ESKLQKLAATHVNAGVTDDQFEIVKEAI
Encalypta_streptocarpa_2057634_KEFD               78 NPKVKNHARFVFMMTGDAAVQLGEKGAFQVL-DSKLQKLATTHINAGVTDDQFEIVKEAI
Ceratodon_purpureus_AF309562                      76 NPKVKNHARYVFMMTGDAAVQLGEKGAYQVL-DSKLQKLAATHINAGVTDDQFEIVKEAI
Sphagnum_fallax_Sphfalx08G081200                  63 NAKLKQHATLVLKLTGDAAVQLGEKGALEAL-GPGLKKLATKHVSAGVVDAHFEVVKVAL
Haplomitrium_mnioides_SRR8202192_DN1398          146 NPQLKAHALTVLKLTGDSAVQLGEKGGLDAL-IPTLRELGTTHFTYGILDEHFDVVKFAL
Treubia_lacunosa_SRR8202205_DN24                 155 NPSLKTHALIVFKMTGDSAVQLGEKGALDAL-HPALKDLGKSHVLHGVFDEHFDVVKFAL
Zea_mays_NP_001105819.1                           58 HPKLKAHAVTVFVMACESATQLRSTGDVKVR-EATLKRLGATHARAGVADAHFEVVKTAL
Impatiens_glandulifera_XP_047311801.1             57 NPKLKSHALIVFSMTCKTAIQLRKAGKVTVS-ESTLQSLGANHFKYNVAAEHFEVVRFAL
Zea_mays_NP_001104966.1                           67 NPKLKTHAMSVFVMTCEAAAQLRKAGKVTVR-ETTLKRLGATHLRYGVADGHFEVTGFAL
Impatiens_glandulifera_XP_047313706.1             72 NPKLKPHARTVFVMTCESAVQLRKAGKVVVR-ESSLKKMGSIHHKYGVVSEHFDVTRYAL
Solanum_tuberosum_NP_001275068.1                  58 NPKLKVHAKSILVMTCEAAVQLRKAGKVVVR-DSTLKKIGATHFKYGVADEHFEVTKYAL
Arabidopsis_thaliana_NP_179204.1                  63 NPKLKPHAMSVFVMCCESAVQLRKTGKVTVR-ETTLKRLGASHSKYGVVDEHFEVAKYAL
Liriodendron_tulipifera_YP108A_19G097700          81 NTKLKRHAMSVFVMTCESAVQLRKAGKPTVR-ESSLKNLGATHFKYGVLDEHFEVAKFAL
Liriodendron_tulipifera_YP108A_19G097600         130 NPKLRPHAMSVFVMTCESAVQLRKVGKPTVR-ESSLKDLGATHFKYGVVDEHFEVTKFAL
Ricinus_communis_XP_015574581.2                  142 NPKLKPHAMTVFVMTCESAVLLRKAGKVTVK-ESNLKDLGATHFRYGVVDEHFEVTKFAL
Prunus_avium_XP_021813455.1                       60 NPKLKPHAISVFVMTCESAVQLRKSGKVTVR-ESTLKRLGGVHFKSGVVDEHYEVTKFAL
Ricinus_communis_XP_002519108.2                   63 NPKLKPHAMSVFIMTCESAVQLRKAGKVTVR-ESSLKKLGAVHFKYGVVDEHFEVTKFAL
```

```
                                            241 .......250.......260.......270.......280.......290.......300
Anthoceros_agrestis_OXF_0001161.318         192 LRTIEESLP-GPDTKKVV----AAWSEA--YDELVNIIKAEMQATRSAQT----------
Anthoceros_agrestis_OXF_0000021.326         128 LQTIAGDLP-GPDSADCN----AGRAGGASLGRITAAASKGLQSFLLQDFK-WQIREVGM
Anthoceros_agrestis_OXF_0001161.315         125 LQTIAGELP-GPDAASLT----EAWGEA--EDTLADTMKAEMQAA---------------
Anthoceros_angustus_AANG007930              175 LQTIAEELP-GPDSAELT----EAWGQA--EDTLADIMKAEMQAE---------------
Radula_lindenbergiana_2085089_BNCU          200 LVTLAEGFP-DVWNAELK----EAWATA--YDELVFAIKSEMKIVRERQAVAA-------
Ricciocarpos_natans_2003425_WJLO            216 LQTIQEGLP-ELWSPELK----AAWGQA--YDELTAVIKTEMKAKRAQAAA---------
Marchantia_polymorpha_Mapoly0014s0083.1     118 LHTIEGAVP-ADWTAEQKNATTHAWSRA--YDELVAVVQAEMDATRKAAEAAWRSDY---
Marcantia_paleacea_2082843_LFVP             118 LHTIEGAVP-AEWSAEQKDATTQAWSGA--YDVVVAVVQAEMDATRKAAAAAWKSDY---
Lunularia_cruciata_SRR8202184_DN579         205 LQTLQEALP-NMWSPKLK----AAWALA--YDEIVDVIKLEMKAKKEATPAAVAFRSLD-
Sphaerocarpos_texanus_2004434_HERT          205 LQTIQEGLP-DMWSPELK----AAWAKA--YDVLVDGIKAEMRAKKAADEAVAATA----
Marchantia_polymorpha_Mapoly0104s0018.1     119 LSTIESLLP-EDWPAEKKVATYGAWAQA--YDELAGVVINEMQVVRDAEVASAAEPK---
Marchantia_polymorpha_Mapoly0104s0016.1     119 LSTIESLLP-EDWPAEKKVATCGAWSQA--YDELAGVVINEMQAIRDAEKAPAAEEAPEA
Lunularia_cruciata_SRR8202184_DN152         120 LTTITSLLP-EEPSAEKKEATLGAWATA--EDVLSGQMITEMQAVRDAQEPATA------
Sphaerocarpos_texanus_2040458_HERT          117 LTTIGSLLP-EEWSVEKKEACNGAWAQA--YDELSGAMKAEMQVVRDAQA----------
Medicago_truncatula_1g090810                118 LKTIKKAVE-DKWSEELD----CAWGIA--YDELAAAIKKAMGWP---------------
Arabidopsis_thaliana_NP_187663.1            119 LRTLKEGLG-EKMNEEVE----GAWSQA--YDHLALAIKTEMKQEES-------------
Aquilegia_coerulea_2G156900                 116 LRTIKDGVG-EKWSEELC----GAWSEA--YDQLATAIKTEMKEEAEQINSSSSA-----
Lactuca_sativa_XP_042755330.2               116 LRTVEKGVG-EKWSEEMK----GAWSEA--YDQLAAAILSEMSKEAAETTDEP-------
Lactuca_sativa_XP_023773123.1               116 IRTVEKAMG-EKCSEEMK----SAWCEA--YDHLAAAIKTEMKQEAAQIQTQL-------
Liriodendron_tulipifera_YP108A_18G052100    117 LRTIRAAMG-EKWSVEMG----GAWGEA--YDLLAAAIKAEMKKEEASVAAIKAEMKKEE
Solanum_tuberosum_XP_006364443.1            117 LRTVEEAMG-KKWNDEMK----EAWGEA--YDQLAAAIKAEMHAEAAA------------
Ricinus_communis_XP_048232168.1             116 LRTVQEAIG-EKWNEEMS----GAWGEA--YDQLAAAIKSEMKEEAATAA----------
Physcomitrium_Phpat.026G010400              178 LRTIAAGLP-DLWSPALK----QAWADA--YDALATTLKNEMHAQAAAAAAKSHT-----
Physcomitrium_Phpat.022G055000              137 LYAIEMGVP-DLWSPELK----SAWGDA--YDMLAEQVKAEMHAQRSAATS---------
Encalypta_streptocarpa_2057634_KEFD         137 LYAIEMGVP-ELWSPELK----SAWGDA--YDMLADAVKTEMRAQRAAARRCLV------
Ceratodon_purpureus_AF309562                135 LYAIEMGVP-ELWSPELK----SAWGDA--YDMLAAAVKAEMHAQRAAAQ----------
Sphagnum_fallax_Sphfalx08G081200            122 LKTIKEGLP-ELWSTELN----QAWADA--YDALAGAIKIEMHTQILKAETTGEVETLIT
Haplomitrium_mnioides_SRR8202192_DN1398     205 LNTLKEAFP-EKWTPELK----EAWAVA--YDEVVLVIKTELHAARDNKQAEEHAVAV--
Treubia_lacunosa_SRR8202205_DN24            214 LETIKQALP-DQWSPELH----GAWSDA--YDELVNVIKDEMKITRSSSVAATA------
Zea_mays_NP_001105819.1                     117 LDTIRDAVP-DMWTPEMK----AAWEEA--YDQLAAVIKEEMKNAAAAEEQTKNAATAAE
Impatiens_glandulifera_XP_047311801.1       116 LETIKEAVP-EIWSPELK----AAWGTA--YDELATAIKNEMKPPVLTS-----------
Zea_mays_NP_001104966.1                     126 LETIKEALPADMWSLEMK----KAWAEA--YSQLVAAIKREMKPDA--------------
Impatiens_glandulifera_XP_047313706.1       131 LETIKEAVP-EMWSPEMK----NAWGTA--YDHLVAAIKAEMKSLADDS-----------
Solanum_tuberosum_NP_001275068.1            117 LETIKEASP-EMWSVEMK----NAWGEA--YDQLVSAIKTEMK-----------------
Arabidopsis_thaliana_NP_179204.1            122 LETIKEAVP-EMWSPEMK----VAWGQA--YDHLVAAIKAEMNLSN--------------
Liriodendron_tulipifera_YP108A_19G097700    140 LDTIKEAVP-TMWSPETK----DAWGEA--YDGLVAAIKIEMKPPS--------------
Liriodendron_tulipifera_YP108A_19G097600    189 LETIKEAVP-TMWSSDMK----VAWGEA--YDELVAAIKKEMKPSS--------------
Ricinus_communis_XP_015574581.2             201 LETIKEAVP-EMWSPDMK----NAWAEA--YDQLVTAIKNEMKSPS--------------
Prunus_avium_XP_021813455.1                 119 LETIKEAVP-EMWSPEMK----NAWGEA--YDQLVAAIKAEMKPSP--------------
Ricinus_communis_XP_002519108.2             122 LETIKEAVP-EIWSPEMK----NAWAEA--YDQLVAAIKTEMKPSS--------------

                                            301 .......310.......320.......330....
Anthoceros_agrestis_OXF_0001161.318         235 ------------HPQT------------------
Anthoceros_agrestis_OXF_0000021.326         182 LVICDRRDPA--TPFESWYRSKAPHHIVSSS---
Anthoceros_agrestis_OXF_0001161.315         163 -----REAQA------------------------
Anthoceros_angustus_AANG007930              213 -----RAAQA------------------------
Radula_lindenbergiana_2085089_BNCU              ----------------------------------
Ricciocarpos_natans_2003425_WJLO                ----------------------------------
Marchantia_polymorpha_Mapoly0014s0083.1         ----------------------------------
Marcantia_paleacea_2082843_LFVP                 ----------------------------------
Lunularia_cruciata_SRR8202184_DN579             ----------------------------------
Sphaerocarpos_texanus_2004434_HERT              ----------------------------------
Marchantia_polymorpha_Mapoly0104s0018.1         ----------------------------------
Marchantia_polymorpha_Mapoly0104s0016.1     176 PAA---ESKA--LP--------------------
Lunularia_cruciata_SRR8202184_DN152             ----------------------------------
Sphaerocarpos_texanus_2040458_HERT              ----------------------------------
Medicago_truncatula_1g090810                    ----------------------------------
Arabidopsis_thaliana_NP_187663.1                ----------------------------------
Aquilegia_coerulea_2G156900                     ----------------------------------
Lactuca_sativa_XP_042755330.2                   ----------------------------------
Lactuca_sativa_XP_023773123.1                   ----------------------------------
Liriodendron_tulipifera_YP108A_18G052100    170 -ACSAAAIKAEMKKEEACSAAAIKAEMKKEEAST
Solanum_tuberosum_XP_006364443.1                ----------------------------------
Ricinus_communis_XP_048232168.1                 ----------------------------------
Physcomitrium_Phpat.026G010400                  ----------------------------------
Physcomitrium_Phpat.022G055000                  ----------------------------------
Encalypta_streptocarpa_2057634_KEFD             ----------------------------------
Ceratodon_purpureus_AF309562                    ----------------------------------
Sphagnum_fallax_Sphfalx08G081200            175 KPETTGEVETLITKPE--TTGEVETLITKPETA-
Haplomitrium_mnioides_SRR8202192_DN1398         ----------------------------------
Treubia_lacunosa_SRR8202205_DN24                ----------------------------------
Zea_mays_NP_001105819.1                     170 ETTNAAAAEEETTNA----AAAVDAS--------
Impatiens_glandulifera_XP_047311801.1           ----------------------------------
Zea_mays_NP_001104966.1                         ----------------------------------
Impatiens_glandulifera_XP_047313706.1           ----------------------------------
Solanum_tuberosum_NP_001275068.1                ----------------------------------
Arabidopsis_thaliana_NP_179204.1                ----------------------------------
Liriodendron_tulipifera_YP108A_19G097700        ----------------------------------
Liriodendron_tulipifera_YP108A_19G097600        ----------------------------------
Ricinus_communis_XP_015574581.2                 ----------------------------------
Prunus_avium_XP_021813455.1                     ----------------------------------
Ricinus_communis_XP_002519108.2                 ----------------------------------
```

```
                                         1 ........10........20........30........40........50........60
Mesotaenium_endlicherianum_247           1 ------------------------------------------------------------
Mesotaenium_endlicherianum_245           1 ------------------------------------------------------------
Coleochaete_orbicularis_comp21145        1 MASTLQ------------------------STTLTAGR------------IASG------
Coleochaete_orbicularis_comp29760        1 ------------------------------------------------------------
Coleochaete_orbicularis_comp33470        1 ------------------------------------------------------------
Sphagnum_fallax_Sphfalx08G081200         1 ------------------------------------------------------------
Physcomitrium_Phpat.026G010400           1 ---------------------------------MSGSHSK--------------------
Physcomitrium_Phpat.022G055000           1 ------------------------------------------------------------
Encalypta_streptocarpa_2057634_KEFD      1 ------------------------------------------------------------
Ceratodon_purpureus_AF309562             1 ------------------------------------------------------------
Selaginella_selaginoides_2042860         1 ------------------------------------------------------------
Selaginella_kraussiana_2006219           1 -----------------------------------------------------------M
Selaginella_lepidophylla_2003383         1 -----------------------------------------------------------M
Selaginella_moellendorffii_80203         1 ------------------------------------------------------------
Anthoceros_agrestis_OXF_000116l.318      1 MPLIALLVTFGEEEGARSK------CPKIL------------------------------
Anthoceros_agrestis_OXF_000002l.326      1 ------------------------------------------------------------
Anthoceros_agrestis_OXF_000116l.315      1 ------------------------------------------------------------
Anthoceros_angustus_AANG007930           1 ------------------------------------------------------------
Radula_lindenbergiana_2085089            1 MAGIAVPQFRP----------------------PAAGVGTT-------NATLPSRGSARL
Ricciocarpos_natans_2003425_WJLO.        1 MASMNLSRTLATR---ANW------CTTAALDSLRGSWSTT-------TATVTPQVSMRR
Marchantia_paleacea_HB                   1 ------------------------------------------------------------
Marchantia_polymorpha_HBa                1 ------------------------------------------------------------
Lunularia_cruciata_SRR8202184_DN579      1 MAYVNLASRVATR---APF------CTNTGRTTTSKVTTRA-------ADDA-----F--
Sphaerocarpos_texanus_2004434            1 MACVKFASNVGAR---GSL------MTPFDTCHPSRSSGAV-------YAIQ-----FPL
Marchantia_polymorpha_HBb                1 ------------------------------------------------------------
Sphaerocarpos_texanus_2040458            1 ------------------------------------------------------------
Lunularia_cruciata_SRR8202184_DN152      1 ------------------------------------------------------------
Dipteris_conjugata_2105411               1 MDAISANP--------AHFAVSAK----------------LGDAIGSHGLCASPRSR---
Ceratopteris_richardii_19G045800         1 ---MPFS----------IMETKSA-----------------IQAASLPNICVDHLKSW--
Ceratopteris_richardii_19G045900         1 ---MPFP----------NMETKSA-----------------IQAASSPSICVNHSKSW--
Pteris_vittata_2100976                   1 MQS---I----------NT----------------------IQPSSTNIVYASQLKSW--
Gaga_arizonica_2011202                   1 MQA---I----------NR----------------------I----QNNVYASHVKAW--
Alsophila_spinulosa_34116                1 MEAVISP----------NFVHTGA-----------------SCGVAGKGYYASHGKAW--
Plagiogyria_japonica_2016413             1 MEAVISA----------NFVQTGS-----------------YGL---GITHASHVKAW--
Equisetum_giganteum_18572                1 MEAIART---------NA------------TCPVLSGNMSKSG-----------------
Phylloglossum_drummondi_2018578          1 ------------------------------------------------------------
Sceptridium_dissectum_2085970            1 MDAAAAV--------AWNF---RCCPTRIPGRDLSSSTSTRLGGPF------QH-ISF--
Arabidopsis_HB2                          1 ------------------------------------------------------------
Gossypium_arboreum_HB2                   1 ------------------------------------------------------------
Nelumbo_nucifera_HB2a                    1 ------------------------------------------------------------
Nelumbo_nucifera_HB2b                    1 ------------------------------------------------------------
Haplomitrium_mnioides_398                1 MASRIVATTTHSLPAG---------Y----GGCWSQSS-ESY------G----AVARV--
Treubia_lacunosa_SRR8202205              1 MAGVTAQ---AL--------------GLQTSMPFVISS-RTVGGRTTTSRLDSSQKGV--
Gnetum_montanum_2055691_GTHK             1 MDAIVCR---AVLHRSA-------------TIPLPVQS-RF-------CVSATFTER---
Zea_mays_HB1b                            1 ------------------------------------------------------------
Parasitaxus_usta_2006444                 1 MTTISMM---AALPVYNNFKGSTHKYGSIEVGS---------------------------
Lagarostrobos_franklinii_2085978         1 ------M---AALSVYNNFTGSTQKYGSIKAGP---------------------------
Manoao_colensoi_2059215                  1 MTTISMM---AALPVYNNFAGSTQKYGSIKLGP---------------------------
Acmopyle_pancheri_2008292                1 MATISMM---AALSAYNNFAGSTQKYGSIKVGN---------------------------
Podocarpus_rubens_2007999                1 MATISMM---TAISTSNNLAGSTWKYGSIKVSP---------------------------
Gingko_biloba_Gb_32168                   1 MAGSSSV---AV------------------------------------------------
Metasequoia_glyptostroboides_2059154     1 MAALSSV---RVFTKSKTT-GFQSAD----GALNSTSR-RT-------GLLATHKVV---
Cryptomeria_japonica_2142292             1 MAALSSV---RVFTKSKAA-GFQS-D----GTLNSISR-RT-------GQLATHKAV---
Chamaecyparis_lawsoniana_2065999         1 MAALSSA---RVFTKSKVA-GFQS-D----GTLNSISR-RT-------GLLATHKAV---
Thuja_plicata_29378600s0009              1 MAALSSA---RVFTKSKVA-GFQS-D----GTFNLISR-RT-------GLMATHKAV---
Cedrus_libani_2003393_GGEA               1 MAAAYSF---TVLPGGMNISATQ--NGTMKGQLLSVA---------------RQ-MEL--
Pinus_jeffreyi_2012080_MFTM              1 ------------------MAPIYQXDGTIKGQLLSVA---------------RH-KDL--
Picea_sitchensis_EF677333                1 MAAVYSI---AVLPRGVNISASQNVHGTIKGQLLPVA---------------RH-MNF--
Picea_glauca_BT116523                    1 MAAVYSI---AVLPRGVNISASQNVHGTVKAQLLPVA---------------RH-MDF--
Picea_abies_MA_32466g0010                1 MAAVYSV---AVLPRGVNISASQNVHGTVKAQLLPVA---------------RH-LDF--
Encephalartos_barteri_2083833            1 MAPAAFM---AVLPGSIAVASQKELS-TTKMYALSIGN-QR--------DHLSHVKVI--
Zea_mays_HB1a                            1 ------------------------------------------------------------
Amborella_trichopoda_HB1                 1 ------------------------------------------------------------
Arabidopsis_HB1                          1 ------------------------------------------------------------
Nelumbo_nucifera_HB1                     1 ------M---HLLSNILASK---------ATLLLSRGT-RT-------CDVAAELSTI--
Parasponia_andersonii_HB1                1 ------------------------------------------------------------
Trema_orientale_HB1                      1 ------------------------------------------------------------
Trema_virgatum_HB1                       1 ------------------------------------------------------------
Trema_tomentosa_HB1                      1 ------------------------------------------------------------
Glycine_max_HB1                          1 ------------------------------------------------------------
Cajanus_cajan_HB1b                       1 ------------------------------------------------------------
Theobroma_cacao_HB1                      1 ------------------------------------------------------------
Pisum_sativum_HB1                        1 ------------------------------------------------------------
Cajanus_cajan_HB1a                       1 ------------------------------------------------------------
```

```
                                      61 ........70........80........90.......100.......110.......120
Mesotaenium_endlicherianum_247         1 -------------------------MAWKNC------FCGGSKAEKE----EEEHNG---
Mesotaenium_endlicherianum_245         1 ------------------------------------------------------------
Coleochaete_orbicularis_comp21145     19 -LGAPGRTSENAG-KKATGLKLNRGLSSSQA------FRGQPLL--------STTAG---
Coleochaete_orbicularis_comp29760      1 -------------------------------------MRREPST--------D-------
Coleochaete_orbicularis_comp33470      1 ------------------------------------------------------------
Sphagnum_fallax_Sphfalx08G081200       1 ------------------------------------------------------------
Physcomitrium_Phpat.026G010400         8 --VRVTQTVAC-------LLPGCVK-------------QRILNFCAL----VVVSLE--E
Physcomitrium_Phpat.022G055000         1 ------------------------------------------------------------
Encalypta_streptocarpa_2057634_KEFD    1 ------------------------------------------------------------
Ceratodon_purpureus_AF309562           1 ------------------------------------------------------------
Selaginella_selaginoides_2042860       1 ----------------------------MAASVMSKLEGN---------DFQATFVGLGR
Selaginella_kraussiana_2006219         2 ASSSRIATQVHMKM-QT---------GWMAKSSWSSRKA-------VHMVPQSM------
Selaginella_lepidophylla_2003383       2 GSTSMIQSGSCIQL-RSSLLPRAAPNGITSSLSVSRAFE-------LRTLRAASSAG--I
Selaginella_moellendorffii_80203       1 ------------------------------------------------MFPLSSKVG--I
Anthoceros_agrestis_OXF_000116l.318   25 -----ISSADCMHG-KHR----ATIHR--RSSAAAQQHRRPGGLRLAHCVH---------
Anthoceros_agrestis_OXF_000002l.326    1 ------------------------------------------------------------
Anthoceros_agrestis_OXF_000116l.315    1 ------------------------------------------------------------
Anthoceros_angustus_AANG007930         1 -----------MTE-TRR----YELGAWAASGVAVAVFGDPRDLCKVLCFSHSLFSV--T
Radula_lindenbergiana_2085089         32 -SLGDTSRSAY--H-QSR----SPALRWTSS----KAFGTQT-FLPERCPFASVHRG---
Ricciocarpos_natans_2003425_WJLO.     45 SVVGSTVKSSSSGE-NRR----HLWLSSTHS----GVFGPSE-LIVR---FASTSSG---
Marchantia_paleacea_HB                 1 ------------------------------------------------------------
Marchantia_polymorpha_HBa              1 ------------------------------------------------------------
Lunularia_cruciata_SRR8202184_DN579   38 -TLPSIRSNNKLSD-RIA----ALKLSSAHS----RGFGDAE-LFHS---FASIS-----
Sphaerocarpos_texanus_2004434         40 GALPSIRSKVF----ARS----TTALSSSQS----KTFGSIE-FFRC---FAATS-----
Marchantia_polymorpha_HBb              1 ------------------------------------------------------------
Sphaerocarpos_texanus_2040458          1 ------------------------------------------------------------
Lunularia_cruciata_SRR8202184_DN152    1 ------------------------------------------------------------
Dipteris_conjugata_2105411            34 -LASRGQTRSC---RL--QARHTHRVSWRDE----SFIGE--RMDTPEYCQL--------
Ceratopteris_richardii_19G045800      29 -SGSRIRHSSC---AAISSLKCARGKSVADI----VFIRPTERSEVSPTSIA--------
Ceratopteris_richardii_19G045900      29 -SGIRIP-SSC---ARISNLKCAGAKSIADV----VSMRPIERRDVFPVSMP--------
Pteris_vittata_2100976                24 -KAIGPFA-----------T-SCKTFRWAAK----CDQGMGGFRSLVEKGSM--------
Gaga_arizonica_2011202                20 -NGGEKRF-----------A-TSTNLRWAAS----NEMRMGSFRSLVEYDKV--------
Alsophila_spinulosa_34116             32 -SG--GGVQ-W---GVIRNGRCLHSLSWARR----EGSHGGRGGLLHCSNKV--------
Plagiogyria_japonica_2016413          29 -SGGQGGVK-W---GV-RNGQCLHFLSWARR----EGRFLRHQGDLF--NKV--------
Equisetum_giganteum_18572             23 -----GMS-NNG--RNHPMSVTMQVFSWKKC----SAIKGLTLG--LGKTIESNG----R
Phylloglossum_drummondi_2018578        1 -------------------------------------------M--AASSTETVE----M
Sceptridium_dissectum_2085970         41 -VAVSSRK-DCVRNFNPSHGHNSGTLSWRNA----AFVGEANLF--YPIFQF-NE----M
Arabidopsis_HB2                        1 ------------------------------------------------------------
Gossypium_arboreum_HB2                 1 ------------------------------------------------------------
Nelumbo_nucifera_HB2a                  1 ------------------------------------------------------------
Nelumbo_nucifera_HB2b                  1 ------------------------------------------------------------
Haplomitrium_mnioides_398             35 -SVS-----NL---RPSRCTGASYNVKWASS----VAFGNLKKV--SAKQFG-VT----S
Treubia_lacunosa_SRR8202205           41 -CAINLRT-NC---TRTQPGSKSKSVSWSRT----AAFGDVNLV--ESSGAY-LT----F
Gnetum_montanum_2055691_GTHK          34 -----LR-------SVPNQARLCSRFYWGRD----LGFTAPTK----------TV----L
Zea_mays_HB1b                          1 ------------------------------------------------------------
Parasitaxus_usta_2006444              31 ---------------LLDTKRRTSGLSWSMT----QRTGESRIL--LSGSLQ-TQ----K
Lagarostrobos_franklinii_2085978      25 ---------------FSDTKQRRSVLSWSIK----QEFGESRIL--LAGSIQ-TL----K
Manoao_colensoi_2059215               31 ---------------LSDTKLRRSGLSWSIT----QVSGECRVL--LSYSLQ-TL----R
Acmopyle_pancheri_2008292             31 ---------------FSHTKQRCSGLSWSIA----QGSGESRIL--LSGSLY-TL----R
Podocarpus_rubens_2007999             31 ---------------LSDTKQRRSGLSWSIT----QRSEESRIL--LSGSFQ-TL----R
Gingko_biloba_Gb_32168                10 -----------------IKGSAILRFQPVEG----SGFAA--IE--IARAEW-FS----M
Metasequoia_glyptostroboides_2059154  42 --LVRSM-------YTNNHNRQNLGLSWKQT----ETFEESKLL--RPKSYR-LS----K
Cryptomeria_japonica_2142292          41 --LVRSM-------YINNQKRQTLGLSWIQT----ETFEESKLL--RPKAHR-LS----K
Chamaecyparis_lawsoniana_2065999      41 --LVRSM-------YTSNQNRQNLGLSRIQT----EIFEESKLF--RPKAHR-LS----K
Thuja_plicata_29378600s0009           41 --LVRSM-------YTNNLNRQNLGLSRIQT----EIFEESKLF--RPKAHR-LS----K
Cedrus_libani_2003393_GGEA            38 -CSM----------KAKPHGGEIIGLQWSRT----QGIRDSQVL--LPTSPF-GL----K
Pinus_jeffreyi_2012080_MFTM           25 -CSL----------KANCDGGQIFGVRWSKS----QRSGDTSIM--LRKSPF-TF----T
Picea_sitchensis_EF677333             40 -CSS----------KASSHGGGIFGLQWSKT----QRYGDSQMM--LRKSPF-IL----T
Picea_glauca_BT116523                 40 -CSS----------KASSHGGGIFGLQWSKT----QRYGDSQMM--LGKSAF-TL----T
Picea_abies_MA_32466g0010             40 -CSS----------KASSHGGGIFGLQWSKT----QRYGDSRMM--LGKSAF-IL----T
Encephalartos_barteri_2083833         46 -SAVAGE-------H--------FGLSESKT----KGFRDRGLV--LKIPH--TF----R
Zea_mays_HB1a                          1 ------------------------------------------------------------
Amborella_trichopoda_HB1               1 -----------------------------------------------------------M
Arabidopsis_HB1                        1 ------------------------------------------------------------
Nelumbo_nucifera_HB1                  33 -CALQGQ-------VSRSRSRHSQELSWAKR----DGSRNA--L--VST-T--TS----L
Parasponia_andersonii_HB1              1 ------------------------------------------------------------
Trema_orientale_HB1                    1 ------------------------------------------------------------
Trema_virgatum_HB1                     1 ------------------------------------------------------------
Trema_tomentosa_HB1                    1 ------------------------------------------------------------
Glycine_max_HB1                        1 ------------------------------------------------------------
Cajanus_cajan_HB1b                     1 ------------------------------------------------------------
Theobroma_cacao_HB1                    1 ------------------------------------------------------------
Pisum_sativum_HB1                      1 ------------------------------------------------------------
Cajanus_cajan_HB1a                     1 ------------------------------------------------------------
```

```
                                            121 .......130.......140.......150.......160.......170.......180
Mesotaenium_endlicherianum_247               23 ----------AVEAKSEAPEAGAGPKAAVTPEEANLLITSWEMVEP-DVNLHAMNFFLRI
Mesotaenium_endlicherianum_245                1 --------------------------MAITAEESQLLQLSWSLIEP-EIQKYSLNFFLRI
Coleochaete_orbicularis_comp21145            60 ----------S--IIPSQGRRERLEVEAFTKMQARLVSDSWEDIKP-NADEHAIRLFSNI
Coleochaete_orbicularis_comp29760             9 ----------------VDSDVGDHSEGHFSPEQAKLVLDSWDTLRK-DLGLHGLRFFSNI
Coleochaete_orbicularis_comp33470             1 --------------------------MALTQEQSKLVGDSWDVMKT-NMEIHGVQLFKNI
Sphagnum_fallax_Sphfalx08G081200              1 --------------------MAEVPSSVFSKDSEILVKQSWELIK-KDTTTNAVIFFTNI
Physcomitrium_Phpat.026G010400               40 P-TMAS--SIGAAPVSVAPETAHGAEKVYSKESVALVKQSWELLK-VDAQANAVAFFKEV
Physcomitrium_Phpat.022G055000                1 ---MAS--AVVNQSEAAAVRAPSKPVKTYSKENEQLVKQSWEILK-KDAQRNGINFFRKV
Encalypta_streptocarpa_2057634_KEFD           1 ---MAP--AIVTPAQAATAPSPAKPAREYSKANEQLVQQSWEILK-KDAQRNGINFFRKV
Ceratodon_purpureus_AF309562                  1 ---MAP--PTVG--TQAQAAPAMVPARVYSKDDEVLVKKSWDILK-KDAQRNGINFFRKV
Selaginella_selaginoides_2042860             24 KGKYPSLTYHKSKELRTSMARASKYEVDENEINEQMIRESWQVLK-KNPQSHAIAFFLKV
Selaginella_kraussiana_2006219               39 --------------------KHGAITAAFTQEHKALVNESWKLLR-KDAPQHAFAFFLKV
Selaginella_lepidophylla_2003383             52 QGRFSRASP------C-KAGYQAARASSESKSNAQLVKDSWQLLR-KDAGTHATNFFLKI
Selaginella_moellendorffii_80203             11 QGILSSAFS------RQPPVCLAVRASAESKSNAELVKDSWRVLK-RDAGTHATAFFLKI
Anthoceros_agrestis_OXF_000116l.318          64 ----LPPPP------TLF---GGLSVRAFSPEQQVLVNESWKTMK-KDAGTVAVKFFLKI
Anthoceros_agrestis_OXF_000002l.326           1 --------M------TTFEGVGVATVGPLSPEKQALVNQSWEQMK-QDAPAVALKFFLKI
Anthoceros_agrestis_OXF_000116l.315           1 -----------------MTTPDVPTVGPLAPEKQALVNQSWDQMK-QDAPGVALKFFLKI
Anthoceros_angustus_AANG007930               43 Q---STESM------TTFEGVGVPTVGPLTPEKQALVSQSWELMK-QDASAVALKFFLKI
Radula_lindenbergiana_2085089                76 --------K------G----RQPWVVRAYTQEQAKLVKDSWKLVQKNDPGKHAVNFFVKI
Ricciocarpos_natans_2003425_WJLO.            89 --------S------GAEGSQPGKVVAAYTREQAELVKSSWKLLK-KDAGKNGLTLFLKV
Marchantia_paleacea_HB                        1 ------------------------MDVNYTKAQAELVKASWEMFK-KDSSGNALIFFTKI
Marchantia_polymorpha_HBa                     1 ------------------------MDVNYSKAEAELVKASWEMFK-KDSIGNALIFFKKI
Lunularia_cruciata_SRR8202184_DN579          79 ---------------HSNTSPANLTVRAYTPEQAELVKSSWQVLK-KDAGNHAHKFFLRV
Sphaerocarpos_texanus_2004434                79 ---------------GSEPSQGTRAVAEFTKEQAALVKETWKLLK-KDAGKNGMTLFLKV
Marchantia_polymorpha_HBb                     1 -----------------------MAASTFTKEQSQLVIESWQMVK-KDSATNSLTFFTKI
Sphaerocarpos_texanus_2040458                 1 -------------------------MASFTPEQSELVIESWQLLK-QDAATHSLTFFTKI
Lunularia_cruciata_SRR8202184_DN152           1 ----------------------MVAPPSFTVEQSQLVIESWQLLK-NDAAKHSLTFFTKI
Dipteris_conjugata_2105411                   74 ---------LS---KSSRHARRALPVRAFTDRQADLVKESWGILRK-DAGNHALAMFLRV
Ceratopteris_richardii_19G045800             73 -----------MRKNSRVREVIAAASASFTKEQAALVKDTWTILKK-DAGRHGMDFFLMV
Ceratopteris_richardii_19G045900             72 ---------ASIYKNSRGSKVIAAALSSFTKEQAALVKDTWAVLKK-DASRHAMDFFLMV
Pteris_vittata_2100976                       59 ---------ALHV-ACNGSNRVAFAMAAFTSEQEALVKDTWAILKK-DAANHGLRLFLTV
Gaga_arizonica_2011202                       55 ---------LSHVVRRKGLNRAAPVRAAFTSKQEALVKDTWAILKK-DAAHHGLALFLTV
Alsophila_spinulosa_34116                    73 ---------GIGRAPDHGSPTTKVTRAAFTEKQEAMVKDTWAILKK-DAGHHALALFLTV
Plagiogyria_japonica_2016413                 69 ---------VVWRAPH----GLARVRGAFTEKQEALVKDTWAILKK-DAGHHAITLFLKV
Equisetum_giganteum_18572                    65 MR--------I--GLRGGAIFRRRSPRAFTECQQTLVQEAWKALKP-NAPQHGLNLFLKV
Phylloglossum_drummondi_2018578              12 AA-----------SSTETVAAGGRSSSSFSEEQAQIVRESWLMLKP-KISDVSINFFLKI
Sceptridium_dissectum_2085970                88 PG----------------FQRRQVCATALTNNQVSLVKTAWDILKK-DAERHGLSLFLKV
Arabidopsis_HB2                               1 -----------------------MGEIGFTEKQEALVKESWEILKQ-DIPKYSLHFFSQI
Gossypium_arboreum_HB2                        1 --------------------------MGFTEKQEGLVKESWEVLKQ-DIPHFSLRFFSLI
Nelumbo_nucifera_HB2a                         1 -------------------------MAGFTEQQEILVKESWETMKQ-NVPELSLRFFTTI
Nelumbo_nucifera_HB2b                         1 -------------------------MSLFTEKQETLVKESWETMKQ-NTPSAYVSSPPKI
Haplomitrium_mnioides_398                    75 SA------------SANAKRGRLSVTTAFTKEQSSLVKESWEALKKDNAGKHALKFFLKI
Treubia_lacunosa_SRR8202205                  85 QSR------------QSSIKDKYLVKNTFTKEQEQLVKKSWAALKK-DSSKHATDFFVRV
Gnetum_montanum_2055691_GTHK                 64 SR------------RSTRPGAASESASSFTEDQEALVKQSWEIMKH-DASEIAFKFFLRV
Zea_mays_HB1b                                 1 --------------------------MGFSEAQEELVLRSWKAMKS-DSESTALKFFLRI
Parasitaxus_usta_2006444                     65 SN--------------GRSRMAKFEIRAFTDEQGTLVKKSWDAMKP-NAPELGLKFFLRV
Lagarostrobos_franklinii_2085978             59 SN--------------GRNRIAKFETRAFSEEQGTLVNKSWDAMKP-NAPELGFKFFLRV
Manoao_colensoi_2059215                      65 SN--------------DRNRIAKFETRAFSEEQGTLVKKSWDAMKP-NAPELGFKFFLRV
Acmopyle_pancheri_2008292                    65 TN--------------GRYRMTKFETRAFSEEQGALVKKSWDAMKP-NAPELGLKFFLKV
Podocarpus_rubens_2007999                    65 SN--------------GRNRMAKFETRAFSEEQGALVKGSWDAMKP-NAPELGLKFFLKV
Gingko_biloba_Gb_32168                       40 --------------VAHNPLYIQLMIRAFSDEQEALVQNSWNVMKK-NAGELGFKLFLMV
Metasequoia_glyptostroboides_2059154         82 SR--------------KYGTAVNLQILAFSEEQGVLVKKSWNVMKK-NAGELGFTFFLRI
Cryptomeria_japonica_2142292                 81 CKRC----SVQIYSAKRYGTSVDLQVQVFSDEQGVLVKKSWNAMKK-NAGELGFTFFLRI
Chamaecyparis_lawsoniana_2065999             81 SR--------------RNGTAVNLQILAFSDEQGVLVKKSWKAMKK-NAGQLGFTLFLRI
Thuja_plicata_29378600s0009                  81 CR--------------RYNTAVNLQILAFSAEQGVLVKKSWKAMKK-NAGELGFAFFLRI
Cedrus_libani_2003393_GGEA                   76 SVKR----SRFG--PAKGRQITNVQIRAFSEEQETLVKQSWNRMKA-NAPELGFKFFLRV
Pinus_jeffreyi_2012080_MFTM                  63 SSNW----SRGI--VSRRSRITNVRATAFTEEQEALVKKSWNAMKS-NAPELGFRFFLRV
Picea_sitchensis_EF677333                    78 SGNG----SRVI--SGKRSRSTNVQTRAFSEEQEALVKKSWNAMKP-NAPQLGFKFFLRV
Picea_glauca_BT116523                        78 SGNG----SRVI--NGKRSRSTNVQTRAFSEEQEALVKKSWNAMKP-NAPQLGLKFFLRV
Picea_abies_MA_32466g0010                    78 SGNG----SRVI--SGKRSRSTNVQTRAFSEEQEALVKKSWNAMKP-NAPQLGFKFFLRV
Encephalartos_barteri_2083833                78 SSS-------------RSSGAKLQAIRALSEEQEVLVKKSWNAMKK-NAGELGFKFFSRV
Zea_mays_HB1a                                 1 ----------------MALAEADDGAVVFGEEQEALVLKSWAVMKK-DAANLGLRFFLKV
Amborella_trichopoda_HB1                      2 QGI-------------KSKGKSIGVIRAFSEEQEALVVESWKVMKK-DAAELGLKFFEKI
Arabidopsis_HB1                               1 --------------------MESEGKIVFTEEQEALVVKSWSVMKK-NSAELGLKLFIKI
Nelumbo_nucifera_HB1                         70 IIN-------------SVERSRMLSVKAFTEEQEALVVKSWNAMKK-DAGELGMKFFLRV
Parasponia_andersonii_HB1                     1 -------------------MSSSEVNKVFTEEQEALVVKAWAVMKK-NSAELGLQFFLKI
Trema_orientale_HB1                           1 -------------------MSSSEVDKVFTEEQEALVVKSWAVMKK-NSAELGLKFFLKI
Trema_virgatum_HB1                            1 -------------------MSSSEVDKVFTEEQEALVVKSWAVMKK-NSAELGLKFFLKI
Trema_tomentosa_HB1                           1 -------------------MSSSEVDKVFTEEQEALVVKSWAVMKK-NSAELGLKFFLKI
Glycine_max_HB1                               1 -----------------------MEGKGFTEEQEALVVKSWNEMKK-NSQELGLKFFKKI
Cajanus_cajan_HB1b                            1 -----------------------MEARVFTEEQEALVVKSWNAIKN-NSQELGLKFFNKI
Theobroma_cacao_HB1                           1 --------------------MTTYEGKVFTEEQEALVVKSWSVMKK-NAAELGLKFFLKI
Pisum_sativum_HB1                             1 --------------------MSTLDRKGFTEEQEALVIKSWSAMKK-NSAELALKFFLKI
Cajanus_cajan_HB1a                            1 ---------------------MTTLPRGFTEEQEALVVKSWTVMKK-NSGELGLKFFLKI
```

```
                                         181 .......190.......200.......210.......220.......230.......240
Mesotaenium_endlicherianum_247            72 FEIAPQATGLFSFLKDS-KGPLEANVKLQKHAATVLQTAGQAAIKLGDAAAVEEL--VPK
Mesotaenium_endlicherianum_245            34 FEIAPAATGLFTFLDND-TTPLATNIRLQKHALTVMTTVGAAVSILADEEALAEF--IPK
Coleochaete_orbicularis_comp21145        107 FKTAPQAMELFSFLRES-GSPKEAHMKLREHAIAVFKMTGEAAVQLGDKGARQSM--API
Coleochaete_orbicularis_comp29760         52 LEIAPEVRGLFSFLKDY-SGKLEDSPRIRQHARDIFRLTGETAAELAATGEVTKL--EPG
Coleochaete_orbicularis_comp33470         34 FEIAPEAKGLFSFIKDD-GSPVEDNPTLRKHATNVFKTAGETMIKFGEKGEMDSL--QPT
Sphagnum_fallax_Sphfalx08G081200          40 FEIAPAAKDFFPFVKNS-TVPYEQNAKLKQHATLVLKLTGDAAVQLGEKGALEAL--GPG
Physcomitrium_Phpat.026G010400            96 FEIAPAAKGFFSFMQDT-SIPFEDNPKVKFHALQVFKLTGDAAAQLGEKGAYELL--QSR
Physcomitrium_Phpat.022G055000            55 FEIAPGAKAMYSFLRDS-TIPFEENPKVKNHARYVFMMTGDAAVQLGEKGAYQVL--ESK
Encalypta_streptocarpa_2057634_KEFD       55 FEIAPEAKAMYSFLRYS-TIPFEENPKVKNHARFVFMMTGDAAVQLGEKGAFQVL--DSK
Ceratodon_purpureus_AF309562              53 FEIAPGAKSMYSFLRDS-TIPFEENPKVKNHARYVFMMTGDAAVQLGEKGAYQVL--DSK
Selaginella_selaginoides_2042860          83 FEISPTSKQLFSFLKDS-EVPIEKNKDLKAHALTVFTLTCEAALNLGKDPSLTAL--KPK
Selaginella_kraussiana_2006219            78 FEIAPTAKQLFSFLRDT-DVPVEKNPHLKSHALMVFTMTCEAALLLGEDPTLAAM--KPK
Selaginella_lepidophylla_2003383         104 FEIAPTVKQLFSFLRNS-TLPVEKNPNLKSHALVVFSMTCEAAVLLGEDPTLEAL--RPK
Selaginella_moellendorffii_80203          64 FEIAPTAKQLFSFMRDS-TVPAEANPHLKSHALVVFTMTCEAAILLGEDPTLAAL--RPK
Anthoceros_agrestis_OXF_000116l.318      110 FEIAPTAKKLFSFIRDS-DVPLEKNPQLKTHALLVLKTTGDAATQLGQKGALEAL--RPV
Anthoceros_agrestis_OXF_000002l.326       46 FEIAPGAIALFSFLKDS-KVPLEQNPRLKVHALLVLKTFGEAATQLGKEGALDEL--TPV
Anthoceros_agrestis_OXF_000116l.315       43 FEIAPGAIALFSFLKDS-KVPLEQNPRLKVHALLVLKTFGEAATQLGKEGALEEL--TPV
Anthoceros_angustus_AANG007930            93 FEIAPGAIALFSFLKDS-KVPLEQNPRLQVHALLVLKTFGEAATRLGKEGALDEL--TPV
Radula_lindenbergiana_2085089            118 FEIAPTVKKMFSFMRDF-SGPMEKNPKLKIHALQVFTLTGQSAAELSEKGGLNVL--LPK
Ricciocarpos_natans_2003425_WJLO.        134 FEIAPSVTQMFSFLRDS-SIPLEQNSQLKAHALTVFKMVGDAAAQLGEKGGLDAL--RAT
Marchantia_paleacea_HB                    36 FEMAPDMRDLFPFTQDP-TLPLEKNYKLKAHALLVFKLTGDAAVQLGENGSIQSL--HPK
Marchantia_polymorpha_HBa                 36 FEVAPDMRDLFPFTQDY-TLPLEKNYKLKAHALLVFKLTGDAAVQLGEHGSIESL--HPK
Lunularia_cruciata_SRR8202184_DN579      123 FEIAPSAAQLFSFLRDS-PVPIEQNPHLKTHALIVFKLTGDAAAQLGEKGALDAL--RPK
Sphaerocarpos_texanus_2004434            123 FEIAPSAQQLFSFLKDS-KVPLEENVQLKGHALNVFKMTGDAAAQLGEKGALDAL--HPK
Marchantia_polymorpha_HBb                 37 FEIAPGARALFSFLKDS-DVPFDKNPKLKAHALNVFRLTGDSASMLGEKGAIDAL--HPK
Sphaerocarpos_texanus_2040458             35 FEIAPGAKALFSFLKDS-DAPLDKNPKLKAHALNVFRLTGDSASMLGEKGAIDAL--HPK
Lunularia_cruciata_SRR8202184_DN152       38 FEIAPGAKALFSFLKDS-SAPLDKNPKLKAHALTVFRLTGDSASMLGEKGAIDAL--HPR
Dipteris_conjugata_2105411               121 FDIAPSAKRLFSFLGESNEVPLEKNPQLKSHALKVFVIICEAATSLKNKGEVSTP--GST
Ceratopteris_richardii_19G045800         121 FDIAPTAKKLFSFLKDS-KEPLEKNSKLKAHALQVFVIICESAASLSSTGAVSTP--GST
Ceratopteris_richardii_19G045900         122 FDVAPAAKKLFSFLKDS-DEPLEKNAKLKAHALQVFVIICESAASLSSTGMVSTP--GST
Pteris_vittata_2100976                   108 FEIAPAAKKLFSFLKDS-NVPLEKNPKLKAHALQVFVIICEAASSLSKKGQVLTP--GST
Gaga_arizonica_2011202                   105 FEIAPAAKKLFSFLKDS-DVPLEKNQKLKAHALQVFVIICEAASSLSKKGQVLTP--GST
Alsophila_spinulosa_34116                123 FEIAPSSKRLFSFLKDS-DVPLEKNPKLKSHALTVFVIICEAATSLRKKGEVSTP--GST
Plagiogyria_japonica_2016413             115 FEIAPSAKRLFSFLKDS-DVPLEKNPLLKSHALTVFVIICEAATSLRKTGEVSTP--GST
Equisetum_giganteum_18572                114 FEIAPSAKKYFSFLSDT-NVPLEKNPKLKAHALLVFTLTCEAAASLLSKDGVSTP--GTS
Phylloglossum_drummondi_2018578           60 FEIAPAAKELFSFLKDS-KVPIELNPRARAHALKVFKMTCECAMELGERGSLDAM--TPT
Sceptridium_dissectum_2085970            131 FEIAPSTKRLFSFLQDS-DVPLAKNPHLKSHALLVFRLTCEAAVSLGNKGEVKTP--GST
Arabidopsis_HB2                           37 LEIAPAAKGLFSFLRDSDEVP-HNNPKLKAHAVKVFKMTCETAIQLREEGKVVVA--DTT
Gossypium_arboreum_HB2                    34 LEIAPGAKNMFSFLRESEEIP-QNNPKLKAHAVKVFKMTCESAIQLREKGEVVVA--DTT
Nelumbo_nucifera_HB2a                     35 LEIAPAAKGMFSFLKDSDEIP-QNNPRLKAHAVKVFKMTCESAVQLREKGEVVVA--ENT
Nelumbo_nucifera_HB2b                     35 LEIAPAAKNMFSFLKDSNELP-QNNPRLKAHAVKVFKMTCESAVQLREKGEVVVA--ENT
Haplomitrium_mnioides_398                123 FEIAPSVQRLFPFLRDS-SVPVEKNPQLKAHALTVLKLTGDSAVQLGEKGGLDAL--IPT
Treubia_lacunosa_SRR8202205              132 FTIAPSAKRLFSFLRDS-SVPLDQNPSLKTHALIVFKMTGDSAVQLGEKGALDAL--HPA
Gnetum_montanum_2055691_GTHK             111 FEIAPSAQNLFSFLRNS-DVPLHKNPKLKAHALTVFKMTCEAAVQLRESGVT-VT--DSS
Zea_mays_HB1b                             34 FEIAPGAKQMFSFLRDAGDAPLEKHPKLKAHAVTVFVMACESATQLRSTGDVKVR--EAT
Parasitaxus_usta_2006444                 110 FEIAPSAKRLFSFLRDS-DIPLEKNANLRAHALTVFKMTCESAVQVGEKGQLRLP--ESN
Lagarostrobos_franklinii_2085978         104 FEIAPSAKRLFSFLRDS-DVPIEKKAKLKAHALTVFKMTCESAVQLGETGQLTLP--ESN
Manoao_colensoi_2059215                  110 FEIAPSAKKLFSFLRDS-DVPLEKNAKLKAHALTVFKMTCESAVQLGETGQLTLP--ESN
Acmopyle_pancheri_2008292                110 FEIAPSAKRLFSFLRDS-DIPLEKNAKLKAHALTVFKMTCESAVQLGEKGQLTVP--ESN
Podocarpus_rubens_2007999                110 FEIAPSAKQLFSFLRDS-DVPLEKNAKLKAHSLTVFKMTCESAVQLGEKGQLTVP--ESN
Gingko_biloba_Gb_32168                    85 FEIAPSAKRLFSFLRDS-DVPIHQNAKLKAHALVVFKMTCESAVLLRKNGRVALPEPESN
Metasequoia_glyptostroboides_2059154     127 FEIAPSAKRLFSFLQNS-EVPLEKNSQLKAHALSVFTLTCESAVQLAESGSITVP--ESI
Cryptomeria_japonica_2142292             136 FEIAPSAKRLFSFLQDS-EVPLEKNSQLKAHALSVFTMTCESAVQLAESGSIRMP--EST
Chamaecyparis_lawsoniana_2065999         126 FEIAPSAKRLFSFLQDS-EVPLEKNPQLKAHALSVFTMTCESAVQLAETGSITVP--EST
Thuja_plicata_29378600s0009              126 FEIAPSAKRLFSFLKDS-EVPLEKNPQLKAHALSVFTMTCESAVQLAESGSIRVP--EST
Cedrus_libani_2003393_GGEA               129 FEIAPSAKRLFSFLQDS-DVPIEKNAKLKAHALTVFKMTCESAVQLREKGTVTFS--EST
Pinus_jeffreyi_2012080_MFTM              116 FEIAPSAKRLFSFLHDS-DVPIEKNGKLKAHALTVFKMTCESAVQLREKGTVTFS--ESN
Picea_sitchensis_EF677333                131 FEIAPSAKRLFSFLQDS-DVPIEKNPKLKAHGLIVFKMTCESAVQLREKGTVTFS--ESN
Picea_glauca_BT116523                    131 FEIAPSAKRLFSFLQDS-DVPIEKNPKLKAHGLIVFKMTCESAVQLREKGTVTFS--ESN
Picea_abies_MA_32466g0010                131 FEIAPSAKRLFSFLQDS-DVPIEKNPKLKAHGLIVFKMTCESAVQLREKGTVTFS--ESN
Encephalartos_barteri_2083833            124 FEIAPSAKRLFSFLRDT-EVPLDKNAKLKAHALTVFTMTCESAVQLREKGTVTFA--GSN
Zea_mays_HB1a                             44 FEIAPSAKQMFSFLRDS-DVPLEKNPKLKTHAMSVFVMTCEAAAQLRKAGKVTVR--ETT
Amborella_trichopoda_HB1                  48 FEIAPSTKRLFSFLRDS-DVPLQQNPKLRSHALSVFAMTCECAVQLRKSGKVTVR--ESN
Arabidopsis_HB1                           40 FEIAPTTKKMFSFLRDS-PIPAEQNPKLKPHAMSVFVMCCESAVQLRKTGKVTVR--ETT
Nelumbo_nucifera_HB1                     116 FEIAPSAQKLFSFLRGS-DVSLEQNQKLKPHAKTVFVMTCESAVQLRKAGKVTVK--DSS
Parasponia_andersonii_HB1                 41 FEIAPSAKNLFSYLKDS-PVPLEQNPKLKPHATTVFVMTCESAVQLRKAGKATVK--ESD
Trema_orientale_HB1                       41 FEIAPSANNLFSYLKDS-PIPLEQNPKLKPHAMTVFVMTCESAVQFRKAGKVTVR--ESN
Trema_virgatum_HB1                        41 FEIAPSAKNLFSYLKDS-PIPLEQNPKLKPHAMTVFVMTCESAVQLRKAGKVTVR--ESN
Trema_tomentosa_HB1                       41 FEIAPSAKNLFSYLKDS-PIPLEQNPKLKPHAMTVFVMTCESAVQLRKAGKVTVR--ESN
Glycine_max_HB1                           37 LEIAPAAQQLFSFLKDS-TVPLEENPKLKPHAMAVFVMTCESAVQLRKAGKVTVR--ESN
Cajanus_cajan_HB1b                        37 LEIAPAAQQLFSFLKDS-NVPLDQNPKLKPHAMAVFVMTCESAVQLRKAGKVTVR--ESN
Theobroma_cacao_HB1                       40 FEIAPSAQKLFSFLRDS-NVPLDQNPKLKPHAMSVFVMTCESAVQLRKAGKVTVR--ESS
Pisum_sativum_HB1                         40 FEIAPSAQKLFSFLKDS-NVPLEQNTKLKPHAMSVFLMTCESAVQLRKAGKVTVR-ESS
Cajanus_cajan_HB1a                        39 FEIAPSAQKLFPFLRDS-TVPLEQNPKLKPHATSVFVMTCDSAVQLRKAGKVTVR--ESN
```

```
                                      241 .......250.......260.......270.......280.......290.......300
Mesotaenium_endlicherianum_247        129 LVALGERHKGYGVIAAHFAVVKQALLDILEKAFG-SAFTDDIK----NAWSKA--FDILA
Mesotaenium_endlicherianum_245         91 LKDLATRHKGYNVVPAHFAVVKVALLDTLESGCG-FDWTPEMA----QAWSKS--FDILA
Coleochaete_orbicularis_comp21145     164 LQSLGKRHFEYGVQPAHYDILKSALIRTLEEEYG-PRFSPQLR----GAWSQA--FDEMA
Coleochaete_orbicularis_comp29760     109 LIALGERHFAYHTKPEHFAVLKTALLRTLEQGYG-SELSPELK----AAWGTA--YDALA
Coleochaete_orbicularis_comp33470      91 LEALGARHFKYGVQPEHFAVVGQALLKTLEDGFG-DKLTPKLR----AAWAQA--YGEVA
Sphagnum_fallax_Sphfalx08G081200       97 LKKLATKHVSAGVVDAHFEVVKVALLKTIKERLP-ELWSTELN----QAWADA--YDALA
Physcomitrium_Phpat.026G010400        153 LHSLAAKHLSKGVQDAHFEVVKEALLRTIAAGLP-DLWSPALK----QAWADA--YDALA
Physcomitrium_Phpat.022G055000        112 LQKLAATHVNAGVTDDQFEIVKEAILYAIEMGVP-DLWSPELK----SAWGDA--YDMLA
Encalypta_streptocarpa_2057634_KEFD   112 LQKLATTHINAGVTDDQFEIVKEAILYAIEMGVP-ELWSPELK----SAWGDA--YDMLA
Ceratodon_purpureus_AF309562          110 LQKLAATHINAGVTDDQFEIVKEAILYAIEMGVP-ELWSPELK----SAWGDA--YDMLA
Selaginella_selaginoides_2042860      140 IKELGYVHLLHGVRDEHYDVVRYALLDTLKNGLK-EEWNSEKS----RAWGKA--YDALV
Selaginella_kraussiana_2006219        135 LLELGPTHLQTQVRDEHFDVVGFALLETLKEALK-EKWTPEMK----AAWSEA--YSAVA
Selaginella_lepidophylla_2003383      161 LLEMGHTHVVHKVLDEHFDVVKYALLETLKEALK-DEWSPDMK----AAWAEA--YDAVT
Selaginella_moellendorffii_80203      121 LLEMGHTHVVHKVIDEHFDVVKYALLETLKEALK-DEWSPSMK----AAWAEA--YDAVT
Anthoceros_agrestis_OXF_000116l.318   167 LLELGRTHVGHGVIDEHFDVVKYALLRTIEESLP-GPDTKKVV----AAWSEA--YDELV
Anthoceros_agrestis_OXF_000002l.326   103 LQKLGRAHVPRGVIDPHFDVVKAALLQTIAGDLP-GPDSADCN----AGRAGGASLGRIT
Anthoceros_agrestis_OXF_000116l.315   100 LQKLGRAHVPRGVIDPHFDVVKSALLQTIAGELP-GPDAASLT----EAWGEA--FDTLA
Anthoceros_angustus_AANG007930        150 LQKLGRAHVPRGVIDPHFDVVKAALLQTIAEELP-GPDSAELT----EAWGQA--FDTLA
Radula_lindenbergiana_2085089         175 LMELGHSHADYGVLDEHFDVVKYALLVTLAEGFP-DVWNAELK----EAWATA--YDELV
Ricciocarpos_natans_2003425_WJLO.     191 LIDLGDSHLTYGVVNEHFDVVKYAMLQTIQEGLP-ELWSPELK----AAWGQA--YDELT
Marchantia_paleacea_HB                 93 LIDLGKKHLGYGVLLEHFVVVKSALLHTIEGAVP-AEWSAEQKDATTQAWSGA--YDVVV
Marchantia_polymorpha_HBa              93 LIDLGKKHLGYGVLLEHFVVVRTALLHTIEGAVP-ADWTAEQKNATTHAWSRA--YDELV
Lunularia_cruciata_SRR8202184_DN579   180 LVELGQSHILYGVLNEHFDVVKYSLLQTLQEALP-NMWSPKLK----AAWALA--YDEIV
Sphaerocarpos_texanus_2004434         180 LVELGQSHVVHGVMNEHFDVVKFALLQTIQEGLP-DMWSPELK----AAWAKA--YDVLV
Marchantia_polymorpha_HBb              94 LKELGKKHVGYGVIAAHFDVVKSALLSTIESLLP-EDWPAEKKVATYGAWAQA--YDELA
Sphaerocarpos_texanus_2040458          92 LKLLGKKHVGYGVIAAHFDVVKTALLTTIGSLLP-EEWSVEKKEACNGAWAQA--YDELS
Lunularia_cruciata_SRR8202184_DN152    95 LKELGRKHVGYGVIPAHFDVVKTALLTTITSLLP-EEFSAEKKEATLGAWATA--FDVLS
Dipteris_conjugata_2105411            179 MKNMAHSHAVSGVVDEHYDVVKYCLLKTIEEGLPAEKWSEEVK----EAWAEA--YSELV
Ceratopteris_richardii_19G045800      178 MKDMARAHHLAGVVDEHYEVVRYCLLKTIQAGLPAEMWNEDVQ----QAWHDA--YDELV
Ceratopteris_richardii_19G045900      179 MKDMARAHYLAGVVDEHYEVVRYCLLKTIQAGLPAGMWNEDVK----QAWHDA--YDELV
Pteris_vittata_2100976                165 MKDMAHAHVISGVVDEHYDVVRYSLLKTVQAGLPAEMWNEEVK----NAWANA--YNELV
Gaga_arizonica_2011202                162 MKDMAHAHVISGVVDEHYDVVRYSLLKTVQAGLPTTVWNEEVK----NAWGDA--YDELV
Alsophila_spinulosa_34116             180 MKDMANSHILSGVVDEHYEVVKFSLLKTVEKGLPAGMWNDEVK----GAWAEA--YDELV
Plagiogyria_japonica_2016413          172 MKKMAHSHAISGVVDEHYEVVKFSLLKTVEEGLPAGEWNDEVK----GAWAEA--YDELV
Equisetum_giganteum_18572             171 IKQMGHTHFTYGILDEHFDVVKYCVVQVLAEGLPKDMWTTEMK----AAWGEA--YDALA
Phylloglossum_drummondi_2018578       117 LRKLGQSHYVRGVVDVQFEVVKCALLTTLQEALQ-DSWRAETK----DAWGEA--YDQLA
Sceptridium_dissectum_2085970         188 MRDMGIAHVKSNVVDEHFDVLRYALLETVEEGLPAGLWTEEMK----EAWGEA--YDELT
Arabidopsis_HB2                        94 LQYLGSIHLKSGVIDPHFEVVKEALLRTLKEGLGE-KYNEEVE----GAWSQA--YDHLA
Gossypium_arboreum_HB2                 91 LKYLGTVHVKSGVKDPHFEVVKEALIRTIEEAIGEEKWNEEMK----NAWGEA--YDQLA
Nelumbo_nucifera_HB2a                  92 LKNLGSVHLKKGVIDPHFEVVKEALLRTIQEAVGE-KWSEEMK----NAWGEA--YDQLA
Nelumbo_nucifera_HB2b                  92 LKNLGSVHLSKGVIDAHFEVVKEALLTTIEEAVGE-KWSEEMA----GAWGEA--FDQLA
Haplomitrium_mnioides_398             180 LRELGTTHFTYGILDEHFDVVKFALLNTLKEAFP-EKWTPELK----EAWAVA--YDEVV
Treubia_lacunosa_SRR8202205           189 LKDLGKSHVLHGVFDEHFDVVKFALLETIKQALP-DQWSPELH----GAWSDA--YDELV
Gnetum_montanum_2055691_GTHK          167 IEHLGQVHYNYGVVDEHFDVVRFCLLETIKEAVL-DIWTPAMK----EAWTEA--YNQLA
Zea_mays_HB1b                          92 LKRLGATHARAGVADAHFEVVKTALLDTIRDAVP-DMWTPEMK----AAWEEA--YDQLA
Parasitaxus_usta_2006444              167 LKDLGEVHLKSGVMDEHYDVVKFCLLETIKDTLP-NMWSPTMK----SAWEEA--YDRLA
Lagarostrobos_franklinii_2085978      161 LKDMGQVHFKSGVVDEHYDVVKFSLLETIKDALP-NMWSPEMK----SAWEEA--YDILA
Manoao_colensoi_2059215               167 LKDMGQAHFKSVVVDEHYDVVKFALLETIKDALP-NMWSPNMK----SAWEEA--YDILA
Acmopyle_pancheri_2008292             167 LKDMGQAHLKSGVVDEHYEVVKFSLLDTIKDALP-NMWSPNMK----SAWEEA--YDVLA
Podocarpus_rubens_2007999             167 LKDMGQAHLKSGVVDEHYEVVKFSLLETIKDALP-DMWSPNMK----SAWEEA--YDVLA
Gingko_biloba_Gb_32168                144 FKDLGRLHLIYGVVDEHFDVVKYCLLKTIEEAVP-EMWSPSMK----SAWEEA--YTQLA
Metasequoia_glyptostroboides_2059154  184 LKDLGTVHFKSGVVDEHFDVTKYSLLKTIEEAVP-DLWSPQLK----SAWEEA--YCQLV
Cryptomeria_japonica_2142292          193 LKELGTVHFKSGVVDEHFDVTKYALLETIKGAAP-DLWSPQLK----SAWEEA--YSQLV
Chamaecyparis_lawsoniana_2065999      183 LKELGAVHVKSGVVDEHFDVTKYALLMTIEEAVP-DLWSPQLK----SAWEEA--YTQLV
Thuja_plicata_29378600s0009           183 LKELGTVHFKSGVVDEHFDVTGYALLKTIEEAVP-DLWSPQLK----SAWEEA--YSQLV
Cedrus_libani_2003393_GGEA            186 VKDMGRLHFKYGVVDEHFDVVKFSLLETIKDAVP-DMWSPEMK----IAWEEA--YTQLA
Pinus_jeffreyi_2012080_MFTM           173 VKDLGKLHFKYGVVDEHFDVVKFCLLETIKDAVP-DIWSLEMK----TAWDEA--YTQLA
Picea_sitchensis_EF677333             188 AKDMGKLHLTYGVLDEHFDVVKFCLLETIKDAVP-DIWSPEMK----IAWDEA--YTQLA
Picea_glauca_BT116523                 188 AKDMGKLHLTYGVLDEHFDVVKFCLLETIKDAVP-DIWSPEMK----IAWDEA--YTQLA
Picea_abies_MA_32466g0010             188 AKDMGKLHLTYGVLDEHFDVVKFCLLETIKDAVP-DIWSPEMK----IAWDEA--YTQLA
Encephalartos_barteri_2083833         181 VKDLGEVHSKYHVLDEHFDVVKFALLETIEQAVP-EMWCPQMK----DAWTDS--YTQLV
Zea_mays_HB1a                         101 LKRLGATHLRYGVADGHFEVTGFALLETIKEALPADMWSLEMK----KAWAEA--YSQLV
Amborella_trichopoda_HB1              105 LKDLGFTHFKYGIVDEHFDVGKFALLETIKEAIP-TMWTVEMK----DAWAEA--YTQLA
Arabidopsis_HB1                        97 LKRLGASHSKYGVVDEHFEVAKYALLETIKEAVP-EMWSPEMK----VAWGQA--YDHLV
Nelumbo_nucifera_HB1                  173 LKELGAVHFKYGVVPEHFDVVKFALLETIKDAVP-EMWSPEMK----NAWGEA--YDKLV
Parasponia_andersonii_HB1              98 LKRIGAIHFKTGVVNEHFEVTRFALLETIKEAVP-EMWSPEMK----NAWGVA--YDQLV
Trema_orientale_HB1                    98 LKRIGAIHFKNGVVHEHFEVTRFALLETIKEAVP-EMWSPEMK----NAWGEA--YDQLV
Trema_virgatum_HB1                     98 LKRLGAIHFKNGVVNEHFE-TRFALLETIKEAVP-EMWSAEMK----NAWGEA--YDQLV
Trema_tomentosa_HB1                    98 LKRLGAIHFKNGVVNEHFE-TRFALLETIKEAVP-EMWSPEMK----NAWGEA--YDQLV
Glycine_max_HB1                        94 LKRLGATHFKAGVAAEHFEVTKLALLETIKEAVP-EMWSPAMK----NAWEEA--HDQLA
Cajanus_cajan_HB1b                     94 LKKLGATHFKAGVAPEHFEVTKLALVETIKEAVP-EMWSEAMK----NAWEEA--HDQLA
Theobroma_cacao_HB1                    97 LKKLGAVHFKYGVVDEHFEVTKFALLETIKEAVP-EMYTAEMK----NAWGEA--YDRLV
Pisum_sativum_HB1                      97 LKKLGATHFKNGVVDEHFEVTKFALLETIKEAVP-EMWSPDMK----NAWGEA-YDQLV
Cajanus_cajan_HB1a                     96 LKKLGATHFKYGVANEHFEVTKFALLETIKEAVP-EMWSPAMK----NAWGEA--YAQLV
```

```
                                              301 .......310.......320.......330.......340.......350.......360
Mesotaenium_endlicherianum_247                182 GTMLKGMAN---------------------------------------------------
Mesotaenium_endlicherianum_245                144 GVMIKTMTGEE-------------------------------------------------
Coleochaete_orbicularis_comp21145             217 EVITEGEMMAEPHAPLPHFIMSEIFSAFEIR-------PQEHLRESEFE--VAGFLLDTA
Coleochaete_orbicularis_comp29760             162 KVVNEGLAKAARGQG-----TGTDFAPYTIR-------K---------------------
Coleochaete_orbicularis_comp33470             144 NMMKLGLEKGAASHV-----VV--------------------------------------
Sphagnum_fallax_Sphfalx08G081200              150 GAIKIEMHTQILKAETTGEVET--LITK----PETT-GEVETLITKPETTGEVETLITKP
Physcomitrium_Phpat.026G010400                206 TTLKNEMHAQAAAAAAKSHT----------------------------------------
Physcomitrium_Phpat.022G055000                165 EQVKAEMHAQRSAATS--------------------------------------------
Encalypta_streptocarpa_2057634_KEFD           165 DAVKTEMRAQRAAARRCLVX----------------------------------------
Ceratodon_purpureus_AF309562                  163 AAVKAEMHAQRAAAQ---------------------------------------------
Selaginella_selaginoides_2042860              193 SMIKQAKSDEVGGDA----FDI--VR----------------------------------
Selaginella_kraussiana_2006219                188 SIIKDEMHALQAASSSSSAX----------------------------------------
Selaginella_lepidophylla_2003383              214 KIIKDEMHAVRRMEAQVV------------------------------------------
Selaginella_moellendorffii_80203              174 KIIKDEMHAVRRMEVAV-------------------------------------------
Anthoceros_agrestis_OXF_000116l.318           220 NIIKAEMQATRSAQT----------------------HPQT-------------------
Anthoceros_agrestis_OXF_000002l.326           158 AAASKGLQSFLLQDFK-WQIRE--VGMLVICDRRDPATPFESWYRSKAP----HHIVSSS
Anthoceros_agrestis_OXF_000116l.315           153 DTMKAEMQAA----------------------REAQA-----------------------
Anthoceros_angustus_AANG007930                203 DIMKAEMQAE----------------------RAAQA-----------------------
Radula_lindenbergiana_2085089                 228 FAIKSEMKIVRERQAVAAX-----------------------------------------
Ricciocarpos_natans_2003425_WJLO.             244 AVIKTEMKAKRAQAAA--------------------------------------------
Marchantia_paleacea_HB                        150 AVMQAEMDATRKAAAAAWKSDY--X-----------------------------------
Marchantia_polymorpha_HBa                     150 AVMQAEMDATRKAAEAAWRSDY--------------------------------------
Lunularia_cruciata_SRR8202184_DN579           233 DVIKLEMKAKKEATPAAVAFRS--LD----------------------------------
Sphaerocarpos_texanus_2004434                 233 DGIKAEMRAKKAADEAVAATAX--------------------------------------
Marchantia_polymorpha_HBb                     151 GVMINEMQVVRDAEVASAAEPK--------------------------------------
Sphaerocarpos_texanus_2040458                 149 GAMKAEMQVVRDAQAX--------------------------------------------
Lunularia_cruciata_SRR8202184_DN152           152 GQMITEMQAVRDAQEPATA-----------------------------------------
Dipteris_conjugata_2105411                    233 DAIKAEFKAGEEEESMRAAKX---------------------------------------
Ceratopteris_richardii_19G045800              232 REMRSQEPKIPAETEVHSS-----------------------------------------
Ceratopteris_richardii_19G045900              233 REMRSQEPQTPGQ-----------------------------------------------
Pteris_vittata_2100976                        219 RAIKAQEEYAAPTX----------------------------------------------
Gaga_arizonica_2011202                        216 LAIKAQEEYTVLSX----------------------------------------------
Alsophila_spinulosa_34116                     234 LAIKAEGGGPALV-----------------------------------------------
Plagiogyria_japonica_2016413                  226 LAIKAEGEPASSX-----------------------------------------------
Equisetum_giganteum_18572                     225 NAIKSEMRKEKEEILTGTPSM---------------------------------------
Phylloglossum_drummondi_2018578               170 SVMKDEIHTQQVQYAQVLSAAA--VX----------------------------------
Sceptridium_dissectum_2085970                 242 KAIKSEMKSEAATIAAGVGAFI--KX----------------------------------
Arabidopsis_HB2                               147 LAIKTEMKQEES------------------------------------------------
Gossypium_arboreum_HB2                        145 EAIKAEMKNHHDETA---------------------------------------------
Nelumbo_nucifera_HB2a                         145 AAIKAEMKKEAAQDPLAQPST---------------------------------------
Nelumbo_nucifera_HB2b                         145 AAIKAEMKEERRQLSFTWPIAL--DS-LTIN-----------WVWSA--NCNR-------
Haplomitrium_mnioides_398                     233 LVIKTELHAARDNKQAEEHAVA--V-----------------------------------
Treubia_lacunosa_SRR8202205                   242 NVIKDEMKITRSSSVAATA-----------------------------------------
Gnetum_montanum_2055691_GTHK                  220 KSIKNEMKAVX-------------------------------------------------
Zea_mays_HB1b                                 145 AVIKEEMKNAAAAEEQTKNAAT--AAEETTN-----------AAAAEEETTNAAAAVDAS
Parasitaxus_usta_2006444                      220 EAIKSEMKAV--------------------------------------------------
Lagarostrobos_franklinii_2085978              214 EAIKNEMKPV--------------------------------------------------
Manoao_colensoi_2059215                       220 EAIKSEMKPI--------------------------------------------------
Acmopyle_pancheri_2008292                     220 EAIKNETKPV--------------------------------------------------
Podocarpus_rubens_2007999                     220 EAIKNKMKPV--------------------------------------------------
Gingko_biloba_Gb_32168                        197 EAIKSEMKPVX-------------------------------------------------
Metasequoia_glyptostroboides_2059154          237 QAIKNEMKPV--------------------------------------------------
Cryptomeria_japonica_2142292                  246 QAIKNEMKPV--------------------------------------------------
Chamaecyparis_lawsoniana_2065999              236 QAIKNEMKPVQ-------------------------------------------------
Thuja_plicata_29378600s0009                   236 QAIKNEMKPVQ-------------------------------------------------
Cedrus_libani_2003393_GGEA                    239 DAIKSEMKTVX-------------------------------------------------
Pinus_jeffreyi_2012080_MFTM                   226 EAIKSEMKTVQP------------------------------------------------
Picea_sitchensis_EF677333                     241 EAIKSEMKTV--------------------------------------------------
Picea_glauca_BT116523                         241 EAIKSEMKTV--------------------------------------------------
Picea_abies_MA_32466g0010                     241 EAIKSEMKTV--------------------------------------------------
Encephalartos_barteri_2083833                 234 KAIKSEMKPSVX------------------------------------------------
Zea_mays_HB1a                                 155 AAIKREMKPDA-------------------------------------------------
Amborella_trichopoda_HB1                      158 FAIKEEMKPTS-------------------------------------------------
Arabidopsis_HB1                               150 AAIKAEMNLSN-------------------------------------------------
Nelumbo_nucifera_HB1                          226 EAIQKEMKPSSGDS----------------------------------------------
Parasponia_andersonii_HB1                     151 AAIKFEMKPSST------------------------------------------------
Trema_orientale_HB1                           151 AAIKFEVKPSST------------------------------------------------
Trema_virgatum_HB1                            150 AAIKFEVKPSST------------------------------------------------
Trema_tomentosa_HB1                           150 AAIKSEMKPSST------------------------------------------------
Glycine_max_HB1                               147 EAIKSEMKPSD-------------------------------------------------
Cajanus_cajan_HB1b                            147 DAIKSEMNPSA-------------------------------------------------
Theobroma_cacao_HB1                           150 AAIKMEMKACSQAS----------------------------------------------
Pisum_sativum_HB1                             150 DAIKSEMKPSS-------------------------------------------------
Cajanus_cajan_HB1a                            149 DAIKSEMKPSS-------------------------------------------------
```

```
                                      361 ...
Mesotaenium_endlicherianum_247            ---
Mesotaenium_endlicherianum_245            ---
Coleochaete_orbicularis_comp21145     268 ---
Coleochaete_orbicularis_comp29760         ---
Coleochaete_orbicularis_comp33470         ---
Sphagnum_fallax_Sphfalx08G081200      203 ETA
Physcomitrium_Phpat.026G010400            ---
Physcomitrium_Phpat.022G055000            ---
Encalypta_streptocarpa_2057634_KEFD       ---
Ceratodon_purpureus_AF309562              ---
Selaginella_selaginoides_2042860          ---
Selaginella_kraussiana_2006219            ---
Selaginella_lepidophylla_2003383          ---
Selaginella_moellendorffii_80203          ---
Anthoceros_agrestis_OXF_000116l.318       ---
Anthoceros_agrestis_OXF_000002l.326   211 ---
Anthoceros_agrestis_OXF_000116l.315       ---
Anthoceros_angustus_AANG007930            ---
Radula_lindenbergiana_2085089             ---
Ricciocarpos_natans_2003425_WJLO.         ---
Marchantia_paleacea_HB                    ---
Marchantia_polymorpha_HBa                 ---
Lunularia_cruciata_SRR8202184_DN579       ---
Sphaerocarpos_texanus_2004434             ---
Marchantia_polymorpha_HBb                 ---
Sphaerocarpos_texanus_2040458             ---
Lunularia_cruciata_SRR8202184_DN152       ---
Dipteris_conjugata_2105411                ---
Ceratopteris_richardii_19G045800          ---
Ceratopteris_richardii_19G045900          ---
Pteris_vittata_2100976                    ---
Gaga_arizonica_2011202                    ---
Alsophila_spinulosa_34116                 ---
Plagiogyria_japonica_2016413              ---
Equisetum_giganteum_18572                 ---
Phylloglossum_drummondi_2018578           ---
Sceptridium_dissectum_2085970             ---
Arabidopsis_HB2                           ---
Gossypium_arboreum_HB2                    ---
Nelumbo_nucifera_HB2a                     ---
Nelumbo_nucifera_HB2b                     ---
Haplomitrium_mnioides_398                 ---
Treubia_lacunosa_SRR8202205               ---
Gnetum_montanum_2055691_GTHK              ---
Zea_mays_HB1b                         192 ---
Parasitaxus_usta_2006444                  ---
Lagarostrobos_franklinii_2085978          ---
Manoao_colensoi_2059215                   ---
Acmopyle_pancheri_2008292                 ---
Podocarpus_rubens_2007999                 ---
Gingko_biloba_Gb_32168                    ---
Metasequoia_glyptostroboides_2059154      ---
Cryptomeria_japonica_2142292              ---
Chamaecyparis_lawsoniana_2065999          ---
Thuja_plicata_29378600s0009               ---
Cedrus_libani_2003393_GGEA                ---
Pinus_jeffreyi_2012080_MFTM               ---
Picea_sitchensis_EF677333                 ---
Picea_glauca_BT116523                     ---
Picea_abies_MA_32466g0010                 ---
Encephalartos_barteri_2083833             ---
Zea_mays_HB1a                             ---
Amborella_trichopoda_HB1                  ---
Arabidopsis_HB1                           ---
Nelumbo_nucifera_HB1                      ---
Parasponia_andersonii_HB1                 ---
Trema_orientale_HB1                       ---
Trema_virgatum_HB1                        ---
Trema_tomentosa_HB1                       ---
Glycine_max_HB1                           ---
Cajanus_cajan_HB1b                        ---
Theobroma_cacao_HB1                       ---
Pisum_sativum_HB1                         ---
Cajanus_cajan_HB1a                        ---
```
